# Co-Developmental Trajectories of Parental Psychological Distress and Child Internalizing and Externalizing Problems in Childhood and Adolescence: Associations with Self-Harm and Suicide Attempts

**DOI:** 10.1007/s10802-023-01034-3

**Published:** 2023-02-07

**Authors:** Xinxin Zhu, Helen Griffiths, Aja Louise Murray

**Affiliations:** 1 Department of Psychology, University of Edinburgh, 7 George Square, EH8 9JZ Edinburgh, UK; 2 Department of Clinical and Health Psychology, University of Edinburgh, Edinburgh, UK

**Keywords:** Parental psychological distress, Internalizing and externalizing problems, Suicide attempts, Self-harm, Joint trajectories

## Abstract

Growing evidence has suggested that parental mental illness and child internalizing and externalizing problems tend to co-occur and engender risk for adverse child outcomes; however, there is considerable heterogeneity in their joint developmental trajectories. This study aimed to evaluate the joint developmental trajectories of maternal and paternal psychological distress and child internalizing and externalizing problems from early childhood to middle adolescence. Given that suicide and self-harm are major public health issues in adolescence and often occur in the context of other mental health issues, we also examined the association between these joint trajectories and these outcomes in adolescence. Parallel-process latent class growth analysis was applied to 14 years of follow-up data from a large-scale, nationally representative sample of youths participating in the UK’s Millennium Cohort Study (MCS; n = 12,520, 50.9% male). Results showed the best-fitting solution had four trajectory classes: (1) low symptoms, 59.0%; (2) moderate symptoms in children, 22.5%; (3) notable symptoms in fathers, 10.7%; and (4) co-occurring maternal and child symptoms, 7.8%. The trajectory groups differed in their self-harm and suicide attempts in adolescence, underscoring the possible importance of the roles of both parental distress and child problem behaviors processes in these outcomes. Future studies will be valuable to rigorously test the directionality and the respective roles of parents and children in this association. Our findings suggest the need for two-generation mental health intervention programs that are tailored based on co-developmental trajectory group membership.

**Supplementary Information:**

The online version contains supplementary material available at 10.1007/s10802-023-01034-3.

Children of parents with psychological distress are often at a heightened risk of internalizing and/or externalizing problems in childhood and adolescence (e.g., Ahmadzadeh et al., 2021; Hails et al., 2018; Wickersham et al., 2020) and evidence suggests that this reflects a dynamic, reciprocal relation between parental and child mental health (Baker et al., 2020; Sifaki et al., 2021). Previous studies have also documented considerable heterogeneity in the developmental trajectories of childhood and adolescent mental health symptoms (Murray et al., 2020; Patalay et al., 2017) and parental psychological distress (Chae et al., 2020; Giallo et al., 2015). Nevertheless, there is a lack of research that addresses both the inter-connectedness of parental and child mental health issues and the heterogeneity in their developmental trajectories. Such investigations are crucial for a more comprehensive understanding of the associations between child and parent mental health issues and for establishing whether the heterogeneity in their co-development can be summarized into a small number of subtypes, potentially differing in their etiology, outcomes, and indications for interventions. The current study thus used a parallel-process latent class growth analysis (LCGA) approach in a large population-representative study to address this gap and establish whether sub-groups defined by joint child and parental mental health trajectories differed in adolescent self-harm and suicidality outcomes.

Transactional models highlight dynamic transactions or reciprocal influences between children and their parents (Sameroff & Mackenzie, 2003). A small number of empirical studies have evaluated the hypothesized relations between parental psychological distress (e.g., depression) and child internalizing (or emotional) or/and externalizing (or behavioral) problems by using variable-centered approaches (e.g., cross-lagged panel and similar models). Some have found that parental depression and child internalizing and/or externalizing problems are concurrently and prospectively related to each other during childhood (Baker et al., 2020; Hails et al., 2018) and adolescence (Fanti et al., 2013), while others have suggested that the direction or magnitude of transactional effects differs by developmental stage (Shaw et al., 2016), type of problem behavior (Sifaki et al., 2021), or between parents (Fanti et al., 2013). For example, research has found effects of maternal depression on child conduct problems during the toddler period and early adolescence, with child effects on maternal depression only observed in early childhood (Shaw et al., 2016). Another study indicated that the effects of maternal and paternal depression on children’s problem behaviors were seen more during early childhood and early adolescence, while a reciprocal relation between paternal depression and child internalizing problems during early adolescence was found (Fanti et al., 2013). Collectively, this association (including pathways from parents to child, reverse pathways, or/and both pathways) has been observed to a greater extent in early childhood and early adolescence. This might be because, e.g., these stages of development can be challenging periods characterized by increasing senses of autonomy and puberty, respectively. Other factors, such as the variables/measures and caregivers included, informants on children’s behaviors, statistical methods, samples, and age of children, as well as whether confounding factors are adjusted for, may also have played a role in the different findings across studies. Although uncertainties remain, these findings generally support the notion that parental mental illness and child problem behaviors are likely to co-occur in a dynamical and reciprocal manner.

Using methods such as latent class growth models (or parallel-process LCGA), ample evidence has also indicated the substantial between-family heterogeneity in the (co-)development of youth internalizing and externalizing problems. Identified trajectory groups have included, for example, pure and co-occurring internalizing and/or externalizing problems at high or moderate levels (or with some changes when transitioning to adolescence) and low symptoms (Murray et al., 2020; Patalay et al., 2017; Speyer et al., 2021). Given that previous studies differ considerably in design, analytical strategy, and age span, these findings have themselves been highly heterogeneous in terms of the specific trajectory groups that have emerged. Nevertheless, it can be seen that the heterogeneity of the trajectories from these studies may manifest itself in two primary ways. One is that internalizing and externalizing problems can have favourable or unfavourable trajectories from childhood to adolescence. These favourable or unfavourable trajectories can then vary in symptom levels across developmental stages (e.g., possibly the onset of or increased internalizing problems in the transition to adolescence due to puberty), giving rise to further subtypes such as ‘early’ vs. ‘late’ onset, ‘adolescent-peaking’ or ‘remitting’, which may also differ in overall severity. The second concerns the extent to which internalizing and externalizing problems are co-occurring over time. It is well-established that these issues are commonly associated and both co-occurring and pure internalizing or externalizing problem groups have been identified in the majority of past trajectory analysis studies (Murray et al., 2020; Patalay et al., 2017; Speyer et al., 2021; Shi & Ettekal, 2021). Thus, evaluating externalizing and internalizing co-developmental trajectories in a large representative sample covering a long age span would be beneficial to account for individual heterogeneity.

Analogously, a small number of studies have evaluated variations in trajectories of maternal and paternal distress during the developmental stages of their offspring’s childhood or/and adolescence. For instance, some research has detected stable no/low parental symptoms, high (or moderate) maternal or paternal symptoms trajectory groups (e.g., Giallo et al., 2015; Skipstein et al., 2012). Others have identified trajectory classes of parental distress that were labeled according to the developmental stage of their children, such as a group with high maternal depression during child preschool period only (It should be noted that this study only included the period from pregnancy to children at age 5) (van der Waerden et al., 2015), and decreasing and increasing maternal depression trajectories in their offspring during adolescence (Chae et al., 2020). Similar to child problem behaviors, maternal and paternal distress can have trajectories characterized by minimal (or no) and problematic symptoms (which may vary in onset and severity) over their lifetimes, appear to remain stable but also change, and may be influenced by a variety of factors (e.g., parenting stress, conflict between spouses). However, based on the heterogeneity of existing evidence, it is difficult to draw general conclusions regarding the variations in the development trajectories of parental distress as prior research has varied considerably in terms of the age span assessed.

As well as the need to resolve existing mixed findings to provide further illumination on the developmental trajectories of child and parental mental health symptoms, there is a need to address the lack of studies examining joint trajectories of maternal and paternal distress across childhood and adolescence. Given the inter-connectedness of child and parental mental health, an important gap remains in considering developmental heterogeneity in children’s internalizing and externalizing problems and parental psychological distress. Accordingly, bringing the child and parental mental health trajectory lines of research together to examine their joint developmental trajectories can improve our understanding of family mental health. It can illuminate for example, how different onset, severity, and chronicity of maternal and paternal psychological distress types cluster with different developmental patterns of internalizing and externalizing problems among children. This provides a basis for then exploring differences in risk factors and etiology, treatment responses, and outcomes. Indeed, a key way in which the subgroups emerging from these analyses may be clinically important is in predicting later adverse outcomes. Knowledge of how subgroups differ in their outcomes can inform more finely tuned interventions tailored for children and families with specific joint trajectories, to reduce their risk of developing these outcomes.

In the present study, we consider the outcomes of adolescent suicide attempts (i.e., operationalized as self-harm behaviors engaged in with at least some suicidal intent) and self-harm (i.e., operationalized as deliberate harm to oneself regardless of suicidal intent). It should be noted that there is some debate in the literature regarding self-harm measurement, with many examining suicidal and non-suicidal self-harm (i.e., suicide attempts and non-suicidal self-injury) separately, and others questioning the reliability of measuring a person’s intent during self-harm, particularly in adolescent populations (Brunner et al., 2014; Kapur et al., 2013). Suicidality (e.g., suicide attempts) and self-harm are of particular current public health interest, and they commonly emerge and are highly prevalent among adolescents (see Cha et al., 2018; Gillies et al., 2018, for summaries), and suicide is one of the biggest causes of mortality in this age group (World Health Organization, 2021). Internalizing and externalizing problems are well established predictors of suicidality and self-harm and their developmental trajectories (e.g., age of onset) have previously been linked to differences in the risk of later adverse outcomes (e.g., Odgers et al., 2007). Therefore, assessing children’s problem behaviors from a developmental perspective and focusing on their suicidality and self-harm in adolescence could provide a better understanding of the effect of the early onset and chronicity of these problem behaviors on suicidality and self-harm, which is important for identifying the early signs of suicide and self-harm risk and for suicide prevention and intervention.

Moreover, recent research has suggested an internalizing and externalizing comorbidity hypothesis for the development of suicidality (e.g., Duprey et al., 2020), i.e., their co-occurring elevations may serve as a unique developmental pathway for suicidality compared to internalizing or externalizing problems alone. This has been attributed to the fact that their co-occurrences confer a high-risk combination of impulsive traits and distress. Additionally, previous research has indicated that youth exposed to negative longitudinal patterns of maternal depression (i.e., chronic-severe depression) in their childhood are at a heightened risk of developing suicidal ideation in adolescence, and youth internalizing and externalizing problems might explain this association (Hammerton et al., 2015) examined the effects of both maternal and child symptoms on child suicidal ideation. However, they only assessed maternal depression during childhood (from 18 weeks’ gestation to age 11) and child symptoms at a single time point (at age 15) and did not include paternal distress. Particularly, when investigating the effects of parent-child trajectories of mental health issues on youth suicide and self-harm risk, it is essential to include not only childhood but also the adolescent period. Indeed, previous findings have indicated the strong potential of focusing on parent-child dyads in adolescence for developing appropriate interventions for youth misbehavior (Moretti & Obsuth, 2009). Taking existing literature together, there has been a paucity of studies that have longitudinally assessed both maternal and paternal distress (especially, paternal effects are understudied) as well as child problem behaviors in childhood and adolescence to investigate their combined effects on suicidality and self-harm. This is important as it could provide a comprehensive understanding of developmental pathways of suicide risk from an early stage by incorporating both parent-and child-level risk factors.

Parallel-process LCGA (which is an inherently exploratory and, at least in part, data-driven analytical approach) is a valuable technique for these types of explorations, i.e., examining co-developmental patterns of parent and child mental health issues. This is because it facilitates the inclusion of multiple variables in a single model to examine their co-changes and takes into account the heterogeneity of individual development by summarizing them in terms of a small number of trajectory subgroups. Previous studies have shown the value of parallel-process LCGA and closely related techniques for shedding light on how the co-development of multiple different variables impact on different outcomes. For example, research has indicated that youth with co-occurring internalizing and externalizing problems persisting from childhood and/or adolescence displayed more delinquency, social exclusion (Murray, Nagin et al., 2021), and suicidality (Orri et al., 2018) than individuals with low/pure internalizing or externalizing problems. Accordingly, such analyses can be especially helpful for examining the adolescent outcomes (i.e., suicide attempts and self-harm) of each joint trajectory group of parental distress and children’s problem behaviors, and they may encode key information about these outcomes in order to inform effective suicide intervention and prevention.

## The Current Study

Informed by theory and evidence from developmental psychopathology, family mental health and suicidality frameworks, this study used a parallel-process LCGA to investigate joint developmental trajectories of parental psychological distress and child internalizing and externalizing problems from early childhood to adolescence (ages 3–14), using a nationally representative sample. This study also evaluated how co-developmental trajectory groups are associated with lifetime suicide attempts (reported at age 17) and past-year self-harm (i.e., with or without suicidal intent, reported at ages 14 and 17) in adolescence. This study hypothesized that trajectory classes with (chronically) high levels of maternal and/or paternal psychological distress would be accompanied by elevated levels of internalizing and/or externalizing problems in their children from early childhood to adolescence, which would be associated with greater adolescent suicide attempts and self-harm.

## Method

### Participants

Participants were from the MCS, an ongoing nationally representative cohort study of children born in 2000–2002 in the UK. The latest Sweep was collected when children were approximately 17 years of age (i.e., Sweep 7), with 18,522, 15,590, 15,246, 13,857, 13,287, 11,714, and 10, 625 productive families participating in Sweeps 1–7, respectively. Details of this cohort study design, variables, and demographic information can be found at: www.cls.ioe.ac.uk/mcs. The current study included data on children aged 3, 5, 7, 11, 14, and 17 years (i.e., Sweeps 2–7), because variables of interest were collected at these ages. The analytic sample for the current study was determined by meeting the following two conditions: (1) Data on the mother (including natural, step, and adoptive parents) in a household were provided by a single person across Sweeps 2–6^1^, as were data on the father, and (2) singleton-born children (as twins share genetics and family environments, which increases the dependence between the data) with valid data on maternal and paternal psychological distress and child internalizing and externalizing problems in at least one of Sweeps 2–6 (n = 12,520; 50. % male). The percentage of participants with valid data from at least 1, 2, 3, 4, and 5 waves (i.e., at ages 3, 5, 7, 11, and 14) on each construct and across families is reported in Table S1 of the supplementary materials.

Of the total 12,520 mothers and fathers, 99.7% were biological mothers, 92.5% were biological fathers (see online Supplementary Tables S2 and S3 for parents’ characteristics).

### Measures

#### Internalizing and Externalizing Problems

Child internalizing and externalizing problems were both measured with the parent-reported Strengths and Difficulties Questionnaire (SDQ; Goodman 2001). Parents (Only the parent acting as the main respondent provided the report of their child symptoms. In the majority of families, only one parent’s reports were collected, and more than 90% were reported by mothers) reported their child’s behavior over the previous six months on a three-point scale ranging from 0 (“*not true*”) to 2 (“*certainly true*”). Parent reports of youth problem behaviors were used because youth reports are not available until age 17. Following recommended practice for population-based samples (Goodman et al., 2010), internalizing problems scale includes emotional and peer problems subscales, and externalizing problems scale includes hyperactivity and conduct problems subscales. Each subscale has five items, and scores on both internalizing and externalizing problems scales range from 0 to 20, with higher scores indicating more problems or symptoms. Cronbach’s αs across Sweeps 2–6 ranged 0.60 to 0.77 for internalizing problems, 0.77 to 0.80 for externalizing problems. Previous studies have generally indicated this measure has favorable factor structure, convergent, discriminant and content validity, reliability (Kersten et al., 2016), and can be used to estimate developmental trajectories of these constructs throughout childhood and adolescence (age 5–14) and across sexes (Murray, Speyer et al., 2021).

#### Parental Psychological Distress

Maternal and paternal psychological distress were both assessed by the 6-item Kessler Psychological Distress Scale (K6; Kessler et al., 2003), which asks in the last month how often participants felt depressed, hopeless, restless, fidgety, worthless, or that everything was an effort. Participants (i.e., both parents) answered on a five-point scale ranging from “*none*” to “*all of the time*”. Possible scores range from 0 to 24, with higher scores indicating high levels of psychological distress. For both parents, the K6 showed good reliability (Cronbach αs ranging from 0.86 to 0.89 for mothers and from 0.81 to 0.86 for fathers) in the current analytic sample cross Sweeps 2–6.

#### Outcome Variables: Suicide Attempts and Self-Harm

At age 14, adolescents rated their self-harm by responding to the yes-or-no question (“*In the past year, have you hurt yourself on purpose in any way*?”). At age 17, self-harm was assessed by the item “*during the last year, have you hurt yourself on purpose in any of the following ways*?” and providing a checklist of six self-harm behaviors (i.e., cutting or stabbing, burning, bruising or pinching, taking an overdose of tablets, pulling out hair, and others). At age 17, youth also self-reported their lifetime history of suicide attempts in response to the yes-or-no question, “*Have you ever hurt yourself on purpose in an attempt to end your life*?” These single-item assessments of suicide attempts or self-harm have been widely used in large population-based surveys (e.g., Russell et al., 2019). Thus, age 14 self-harm and lifetime suicide attempts reported at age 17 had a possible value of 0 or 1. In order to maintain consistency with the aforementioned binary outcomes and facilitate interpretation, we categorized age-17 self-harm as a binary outcome (responses were coded as ‘yes’ if participants answered ‘yes’ to any of the six self-harm behaviors provided, otherwise responses were coded as ‘no’). Using youth reports for the outcomes and parent reports for the trajectories mitigates the risk of common rater bias, which could inflate associations between trajectories and outcomes.

### Statistical Analysis

The parallel-process LCGA was employed to evaluate joint trajectories of parental psychological distress and children’s internalizing and externalizing problems. LCGA is similar to growth mixture modelling (GMM) but differs from it in disallowing within-group variation in trajectories. It is sometimes necessary to constrain within-group variation to zero to facilitate estimation, especially for more complex models. Given that our models combine the developmental trajectories of multiple variables and were fit for the purpose of summarizing sets of trajectories to facilitate new insights, we opted for an LCGA approach. The main implication of this choice is that it is likely that more trajectory groups emerge than would using a GMM approach. Models with one to eight latent classes (models with linear only and both linear and quadratic growth) were evaluated, both considering the exploratory nature of this study and model parsimony.

All models were fit using Mplus, with robust maximum likelihood (MLR) estimation. In using MLR, missing data were handled using full information maximum likelihood (FIML) estimation that provides unbiased parameter estimates when data are missing at random (MAR) in Rubin’s (1976) terms (i.e., that the probability of missingness is independent of the missing values and related only to measured variables in the analysis). The unadjusted results (without stratification, clustering, and weighting adjustment) are reported.

Several criteria were used to select an optimal model: (1) the Lo-Mendall-Rubin (LMR) testing whether the superiority of a k-class trajectory model is against a k-1 class trajectory model, with a significant *p*-value suggesting the former is significantly better than the latter, (2) the Akaike’s Information Criterion (AIC), Bayesian Information Criterion (BIC), and sample size adjusted BIC (SaBIC), with lower values indicating better fit, (3) adequate classification diagnostics (entropy).

Having identified an optimal model, we used the automatic Bolck-Croon-Hagenaars procedures (BCH, Asparouhov & Muthén 2014) to distal outcomes into the parallel-process LCGA to test the predictive effects of class membership on lifetime suicide attempts (reported at age 17) and self-harm at ages 14 and 17. Both ages 14 and 17 self-harm were included in the analysis because it allows for maximum use of the data (since self-harm was collected at these ages). This is also because the finding of the associations between joint trajectories of parental distress and children’s symptoms and age 14 self-harm might be helpful for informing self-harm interventions in early to mid-adolescence, although this finding is cross-sectional in nature. The BCH has been recommended for use with both continuous and binary distal outcomes, and compared with other methods, it has the advantage that latent class solutions are not affected by outcome variables (i.e., it avoids shifts in latent classes).

## Results

### Descriptive Statistics

Descriptive statistics and Pearson correlations for the main study variables are provided in online Supplementary Tables S4 and S5, respectively. At most of the ages studied, child internalizing and externalizing problems, and paternal and maternal psychological distress were positively associated with child self-harm at ages 14 and 17 and lifetime suicide attempts (reported at age 17), but the effect sizes were modest (r = .02-0.17).

### Parallel-Process LCGA

**Fig. 1:**
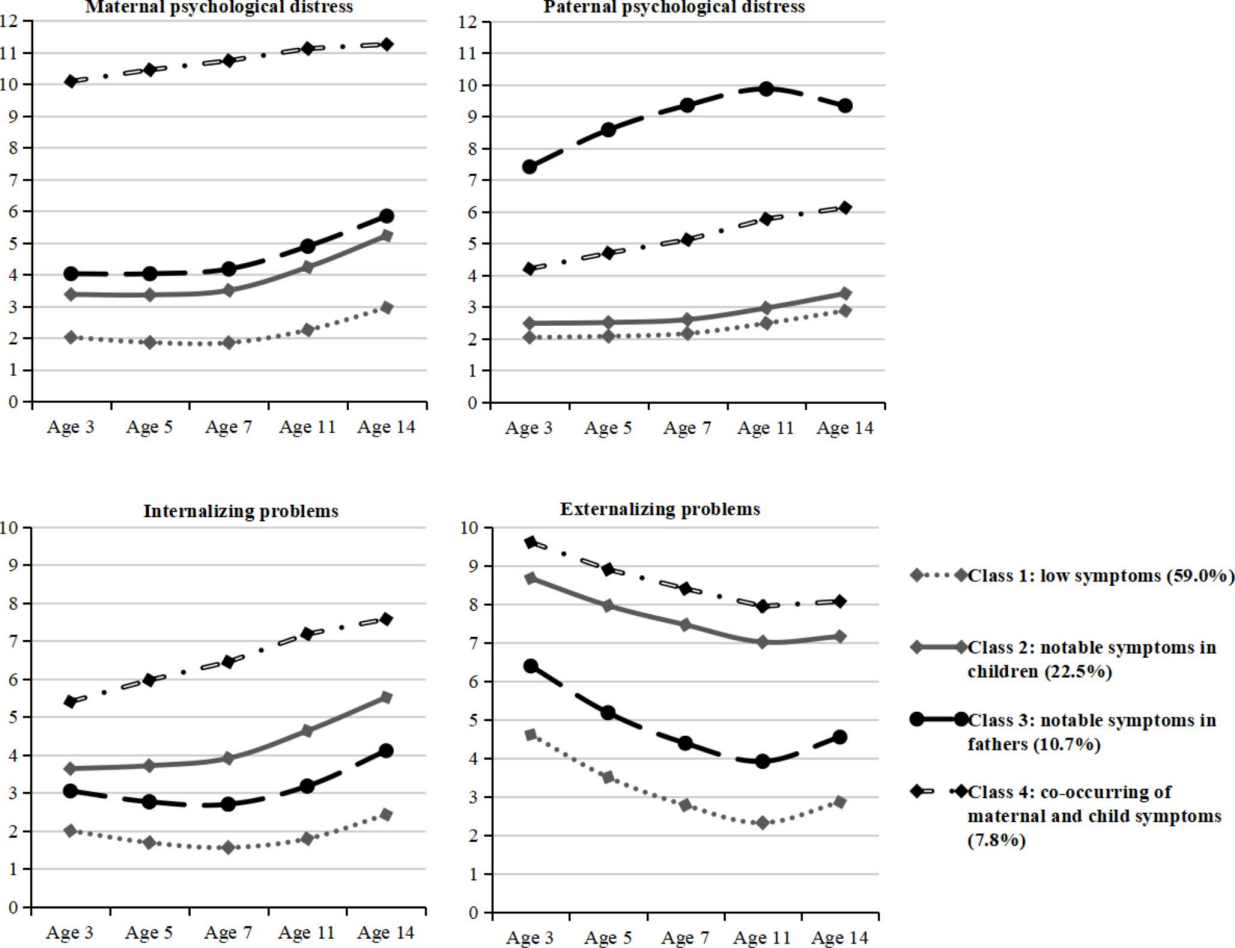
Joint maternal and paternal psychological distress and child internalizing and externalizing problems trajectories (Whole sample)

Fit statistics for the joint trajectory models are provided in online Supplementary Table S6. The four-class model with both linear and quadratic growth was identified as the optimal model based on the fact that LMR-test indicated that the four-class linear and quadratic growth model was better than its three-class counterpart; it has lower AIC, BIC, and SaBIC values than other solutions suggested by the LMR test; and in models with more than six trajectories, the number of participants in some classes was very low. Entropy values were consistently above 0.80, indicating high classification accuracy. Full output for all models is provided at: https://osf.io/q36gp/files/ Growth parameters for the four-class model are provided in online Supplementary Table S7 and visualized in Fig. 1. Trajectory subgroups emerging in the four-class solution basically supported previous findings on developmental trends and patterns of youth problem behaviors (e.g., Speyer et al., 2021) and the association between parental mental health issues and child problem behaviors (e.g., Sifaki et al., 2021). This suggested that the solution was also acceptable on interpretational grounds as well as statistical grounds.

The first trajectory class (59.0% of the sample) was characterized by initially very low and slightly fluctuating maternal and paternal distress and child internalizing problems, and initially moderate-low and decreasing child externalizing problems; thus, it was labeled “low symptoms”. The second class (22.5%) showed initially moderate and increasing child internalizing problems, initially moderate-high and decreasing child externalizing problems, initially moderate-low and increasing maternal distress, and initially low and slightly increasing paternal distress, and was, therefore, labeled “moderate symptoms in children”. The third class (10.7%) was characterized by initially relatively high and increasing, then slightly decreasing paternal distress, initially moderate/moderate-low and increasing maternal distress/child internalizing problems, and initially moderate and decreasing child externalizing problems, which was, thus, labeled “notable symptoms in fathers”. The fourth class (7.8%) showed initially high and slightly increasing maternal distress, initially high-moderate and increasing child internalizing problems, initially high and decreasing child externalizing problems, and initially moderate and increasing paternal distress. This class was, therefore, labeled “co-occurring maternal and child symptoms”. Taken together, even though the mean levels at each age and across each class vary, trajectories of change over time for each type of symptom/reporter/target were fairly consistent.

### Outcomes in Adolescence

Table 1 reports the results of comparisons of adolescent outcomes of suicide attempts and self-harm among the identified trajectory groups. The proportions of youth at age 14 engaged in self-harm in the group of low symptoms (i.e., 0.13) and notable symptoms in fathers (i.e., 0.13) are comparable to the prevalence that was reported in a recent meta-analysis study of self-harm in community-based adolescents (0.13; Gillies et al. 2018). But the proportions of self-harm in the other two groups (i.e., 0.17 and 0.23) at age 14 and in all four groups (i.e., 0.21–0.27) identified at age 17 were somewhat higher than those reported in the aforementioned meta-analysis study. Regarding the proportion of lifetime suicide attempts, except for the co-occurring maternal and child symptoms group (i.e., 0.14), in all other three groups (i.e., 0.05–0.09), it was generally within the range of rates estimated in previous review work in the adolescent population (0.031–0.088; Nock et al., 2008).

**Table 1:** Relations of the four trajectories to outcomes of self-harm and suicide attempts in the whole sample

	Outcome means* (SE) by class				Wald test *p* value					
	Low symptoms (c1)	Moderate symptoms in children (c2)	Notable symptoms in fathers (c3)	Co-occurring maternal and child symptoms (c4)	c1 vs. c2	c1 vs. c3	c1 vs. c4	c2 vs. c3	c2 vs. c4	c3 vs. c4
Age14 self-harm	0.13 (0.01) ^2,4^	0.17 (0.01) ^1,4^	0.13 (0.01) ^4^	0.23 (0.02) ^1,2,3^	0.002**	0.689	< 0.001***	0.064†	0.005**	< 0.001***
Age17 self-harm	0.21 (0.01) ^2,3,4^	0.26 (0.02) ^1^	0.26 (0.01) ^1^	0.27 (0.02) ^1^	< 0.001***	0.004**	0.008**	0.972	0.890	0.878
Lifetime suicide attempts (reported at age 17)	0.05 (0.00) ^2,3,4^	0.09 (0.01) ^1,4^	0.09 (0.01) ^1,4^	0.14 (0.02) ^1,2,3^	< 0.001***	< 0.001***	< 0.001***	0.781	0.005**	0.016*

*Note.* * Outcome means represent endorsement percentage. Numbers in superscript refers to significantly different subgroups in the outcomes. P values indicate for the Wald tests

Youth in the group of co-occurring maternal and child symptoms were more likely to engage in self-harm at age 14 and lifetime suicide attempts (reported at age 17) than those in the other three groups identified in this study, and they also reported more self-harm at age 17 than children in the low symptoms group. Youth in the class of moderate symptoms in children had a higher likelihood of self-harm at age 14, compared to the class of notable symptoms in fathers (though it was only marginally significant) and low symptoms, and at age 17, they reported more past-year self-harm and lifetime suicide attempts than those in the low symptoms group. Youth in the trajectory of notable symptoms in fathers showed greater self-harm at age 17 and lifetime suicide attempts than youth in the low symptoms group.

Supplementary exploratory analyses were conducted to provide additional information into the findings, including sex-stratified analyses; the relation between demographic factors and trajectory class membership; the results adjusted for stratification, clustering, and weighting; and the analyses limited to biological parents (see supplementary materials for details).

## Discussion

This study examined the longitudinal patterns of co-developing parental psychological distress and child internalizing and externalizing difficulties during children’s childhood and adolescence (i.e., ages 3 to 14), using a parallel process LCGA and over 14 years of follow-up data. It also investigated how the identified groups differed in adolescents’ outcomes of suicide attempts and self-harm. Results suggested a four-class solution fitted the data best, and these classes differed in their risk of self-harm and suicide attempts.

Reflecting the normative nature of the sample, the biggest group were not substantially affected by parental or child mental health issues. A small group of families with both mother and child displaying salient and developmentally persistent symptoms (7.8%) was also identified. The presence of this group implies that it is beneficial to consider mothers and children together as a subtype for routine screening and targeted intervention that specifically addresses this interaction. Several mechanisms may explain the co-occurrence between maternal distress and child mental health problems. For instance, maternal distress may put children at the risk of developing problem behaviors due to genetic transmission or chronic exposure to negative emotions (Downey & Coyne, 1990). Further, maladaptive parenting behaviors associated with maternal distress can also increase the risk of developing adjustment problems (Kuckertz et al., 2018). Meanwhile, based on transactional model, children’s adjustment problems might also evoke increases in maternal psychological distress (e.g., Shaw et al., 2016), thus, there might be reciprocal relations between maternal and child problems, or maternal distress might be a consequence of child problem behaviors. Notably, the direction and mechanisms of this association cannot be determined based on the current findings, which address concurrent relations between parental distress and child symptoms over time.

Approximately 23% of children showed moderate/high-moderate internalizing/ externalizing problems in early childhood, and their internalizing and externalizing problems were increasing and decreasing, respectively, as they approached middle adolescence. However, their parents reported low or moderate-low levels of distress, indicating the problems suffered by children in this group were not associated with maternal and paternal distress. Future studies need to investigate and identify correlators that are particularly relevant to the problem behaviors among youth in this class, as it suggests that the correlators may lie primarily outside the family environment.

We also identified a group of families with notable paternal distress (10.7%) whose children only displayed moderate-low or moderate levels of internalizing and externalizing problems. This finding, along with the lack of a trajectory class with both high levels of distress in fathers and internalizing or externalizing problems in children, was in line with previous evidence that the association between paternal mental illness and child difficulties is of small magnitude (e.g., Sifaki et al., 2021). One possible explanation is that mothers, rather than fathers, are typically the primary caregivers, and therefore associations between maternal distress and child problems are more likely to be apparent. However, it should be noted that these small paternal-child associations could also be due to the fact that children’s problem behaviors in this study were mainly reported by mothers.

The four groups differed in their risk of adolescent self-harm and/or suicide attempts. Specifically, youth in the class of co-occurring maternal and child symptoms showed a significantly higher risk of engaging in self-harm at age 14 and lifetime suicide attempts than those in the other three groups. This is consistent with previous findings of a link between both the problematic development of maternal depression across the childhood of their offspring (Hammerton et al., 2015) and following a co-occurring trajectory of internalizing and externalizing problems from childhood to adolescence (Duprey et al., 2020) and a higher adolescent suicide risk. This study adds to this by integrating the co-developmental patterns of children’s internalizing and externalizing problems with longitudinal patterns of parental mental illness to investigate their combined effects. The heightened suicide risk in this group might be due to high levels and persistence of both maternal distress and youth internalizing and externalizing problems and underlying this co-occurring trajectory could be a reciprocal or transactional effect between parental (particularly maternal) and child mental health issues, leading to increased suicidality in adolescence. It also should be noted that there are other possibilities for this association: e.g., the increased suicidality/self-harm might be driven solely by child symptoms, and that maternal symptoms may be an effect of child symptoms (or unrelated to adolescent outcomes of suicidality/self-harm). Moreover, it is plausible that youth suicidality/self-harm could increase parental distress/their own symptoms, given the overlap in assessment time points for distress/symptoms (from age 3 to age 14) and self-harm (at age 14 and 17) and suicide attempts (lifetime suicide attempts reported at age 17).

The group with moderate symptoms in children were at greater risk of developing suicidality and self-harm in adolescence than the low symptoms group. This group and the group with co-occurring maternal and child symptoms had moderate and high comorbidity between internalizing and externalizing problems, respectively, and were also characterized by an elevated risk of self-harm and lifetime suicide attempts during adolescence. These findings are consistent with prior evidence that the co-occurrence of internalizing and externalizing problems over time may be associated with a particularly heightened risk of suicidality in adolescence (Dugré et al., 2020; Duprey et al., 2020). As discussed earlier, this could be due to the fact that externalizing problems may confer impulsivity while internalizing problems confer distress, which when combined, create a high risk for suicidality. The group of notable symptoms in fathers displayed a somewhat higher possibility of self-harm and suicide attempts compared to the low symptoms group. Thus, youth exposed to paternal distress, even when their internalizing/externalizing problems are moderate or low, should receive more attention to reduce their risk of self-harm and suicidality. This finding may suggest paternal effects merit more attention. Particularly, there is a relative dearth of research examining and developing the clinical treatments for paternal psychopathology.

Taken together, our findings indicate that severe maternal psychological distress and children’s internalizing and externalizing problems are inter-related and this is associated with suicidality and self-harm risk in adolescence. This may imply the potential value of screening all family members (especially mothers) for mental health issues if a child or adolescent exhibits emotional or/and behavioral problems. Based on the finding on the co-occurring group, screening and treating both parents’ and children’s mental health issues is not only important for the outcomes of youth suicidality and self-harm but also for youth and parent psychopathology. Existing evidence has suggested that treating only parental psychopathology can benefit the untreated offspring (Pilowsky et al., 2008). Beyond this, the current findings suggest that two-generation interventions addressing both parents’ and children’s problems jointly and their synergistic relationship may be more effective. Specifically, interventions need to attend to both child and parent needs to address this negative child-parent interaction in addition to the overall systemic focus.

Family-based therapies, e.g., family group cognitive-behavioral psychotherapy (e.g., Compas et al., 2009), can be used in two-generation intervention programs that have been shown to be effective in treating child problem behaviors and parental mental illness. Family-focused interventions can also be used to treat youth suicidality and self-harm (Esposito-Smythers et al., 2019; Pineda & Dadds, 2013; Waraan et al., 2022). Additionally, indirect interventions focusing on problems associated with depression, as a new approach, have been shown to be effective in treating depression (Cuijpers, 2021). In this study, for example, an indirect preventative intervention focusing on dynamic parent-child relationships may be a promising direction for having a positive impact on both parental psychological distress and children’s emotional and behavioral difficulties.

### Limitations and Future Directions

This study also has several limitations. First, child internalizing and externalizing problems were mainly reported by mothers, and thus, shared method variance might have inflated the relations between maternal distress and child problem behaviors. This is an important limitation since it may substantially alter the pattern of findings on maternal versus paternal distress in relation to youth symptoms, suicidality, and self-harm, and the current findings should therefore be interpreted with caution. Future studies should replicate the current results by including reports from multiple informants, including fathers and children themselves. Second, due to data availability, self-harm and suicide attempts were only included as outcomes, and self-harm was measured in a broad manner and not specifically as suicidal or non-suicidal self-harm. Future research needs to collect data on youth suicide attempts and explicit measures of non-suicidal self-harm at several time points in order to adjust for prior levels of these variables and avoid overlap between outcomes. In addition, brief measures of parental distress and children’s symptoms were collected, and future research will benefit from detailed measures of both parental and children’s mental health and behavioral issues. Third, although the Strengths and Difficulties Questionnaire (SDQ) has been widely used and has generally demonstrated good psychometric properties, the Cronbach’s αs for the SDQ was low in the MCS study. Fourth, a parallel-process LCGA was applied to analyze the data. Although such analysis can reduce the computational burden (Jung & Wickrama, 2008), it is limited by the assumption of no within-class variability, which can lead to the over-extraction of latent classes compared to growth mixture models (GMMs) that model within-class variability. Fifth, this study might be limited by the potential non-random effect of missing data (e.g., families with a heavier burden of mental illness may be more likely to drop out over and above what is predictable from measured variables) as well as the exclusion of families with single parents and/or parents of the same sex/gender. Additionally, it should be pointed out that although there is a lot of missing data for fathers in MCS, very few cohort studies have very complete fathers’ data (it is not an issue specific to MCS). Fathers’ mental health is a relatively understudied area and future studies should invest in collecting data from fathers. Finally, the present analyses used data from a normative sample and future studies including both normative and clinical samples to examine these processes in high-risk populations will be important.

## Conclusion

In a small number of families (~ 8%), maternal psychological distress and children’s internalizing and externalizing problems persisted concurrently from early childhood to middle adolescence. More than 20% of children displayed moderate internalizing and externalizing behaviors, but their parents did not show mental health issues. Youth in problematic trajectory groups (e.g., the co-occurring maternal and child symptoms group) reported a higher risk of engaging in self-harm and lifetime suicide attempts in adolescence. Together, these findings suggest that two-generation mental health intervention programs could be tailored based on the groups identified in the current study. They indicate that improving the mental health issues of both parents (especially mothers) and children during the offspring’s early developmental stage (i.e., early childhood) may be particularly effective in reducing children’s risk of developing suicidality and self-harm in adolescence.

## Electronic Supplementary Material

Below is the link to the electronic supplementary material.

Supplementary Material 1

## Footnotes

1 This means that relevant data for mothers (and fathers) were provided by different people at different waves, and they were not included in the analysis because it would complicate data matching.

## References

[CR1] Ahmadzadeh, Y. I., Schoeler, T., Han, M., Pingault, J. B., Creswell, C., & McAdams, T. A. (2021). Systematic review and meta-analysis of genetically informed research: associations between parent anxiety and offspring internalizing problems. *Journal of the American Academy of Child & Adolescent Psychiatry*, *60*(7), 823–840. 10.1016/j.jaac.2020.12.037 PMC8259118 33675965

[CR2] Asparouhov, T., & Muthén, B. (2014). Auxiliary variables in mixture modeling: Using the BCH method in Mplus to estimate a distal outcome model and an arbitrary secondary model. *Mplus Web Notes*, *21*(2), 1–22. Scopus.

[CR4] Baker CE, Brooks-Gunn J, Gouskova N (2020). Reciprocal relations between maternal depression and child behavior problems in families served by head start. Child Development.

[CR5] Brunner R, Kaess M, Parzer P, Fischer G, Carli V, Hoven CW, Wasserman D (2014). Life-time prevalence and psychosocial correlates of adolescent direct self‐injurious behavior: a comparative study of findings in 11 european countries. Journal of Child Psychology and Psychiatry.

[CR6] Cha CB, Franz PJ, Guzmán M, Glenn E, Kleiman CR, Nock MK (2018). Annual Research Review: suicide among youth–epidemiology,(potential) etiology, and treatment. Journal of Child Psychology and Psychiatry.

[CR7] Chae HK, East P, Delva J, Lozoff B, Gahagan S (2020). Maternal depression trajectories relate to youths’ psychosocial and cognitive functioning at adolescence and young adulthood. Journal of Child and Family Studies.

[CR8] Compas BE, Forehand R, Keller G, Champion JE, Rakow A, Reeslund KL, Cole DA (2009). Randomized controlled trial of a family cognitive-behavioral preventive intervention for children of depressed parents. Journal of Consulting and Clinical Psychology.

[CR9] Cuijpers P (2021). Indirect prevention and treatment of depression: an emerging paradigm?. Clinical Psychology in Europe.

[CR10] Downey G, Coyne JC (1990). Children of depressed parents: an integrative review. Psychological Bulletin.

[CR11] Dugré JR, Dumais A, Dellazizzo L, Potvin S (2020). Developmental joint trajectories of anxiety-depressive trait and trait-aggression: implications for co-occurrence of internalizing and externalizing problems. Psychological Medicine.

[CR12] Duprey EB, Oshri A, Liu S (2020). Developmental pathways from child maltreatment to adolescent suicide-related behaviors: the internalizing and externalizing comorbidity hypothesis. Development and Psychopathology.

[CR13] Esposito-Smythers C, Wolff JC, Liu RT, Hunt JI, Adams L, Kim K, Spirito A (2019). Family‐focused cognitive behavioral treatment for depressed adolescents in suicidal crisis with co‐occurring risk factors: a randomized trial. Journal of Child Psychology and Psychiatry.

[CR14] Fanti KA, Panayiotou G, Fanti S (2013). Associating parental to child psychological symptoms: investigating a transactional model of development. Journal of Emotional and Behavioral Disorders.

[CR15] Gillies D, Christou MA, Dixon AC, Featherston OJ, Rapti I, Garcia-Anguita A, Christou PA (2018). Prevalence and characteristics of self-harm in adolescents: meta-analyses of community-based studies 1990–2015. Journal of the American Academy of Child & Adolescent Psychiatry.

[CR16] Giallo R, Cooklin A, Brown S, Christensen D, Kingston D, Liu CH, Wade C, Nicholson JM (2015). Trajectories of fathers’ psychological distress across the early parenting period: implications for parenting. Journal of Family Psychology.

[CR17] Goodman A, Lamping DL, Ploubidis GB (2010). When to use broader internalising and externalising subscales instead of the hypothesised five subscales on the Strengths and Difficulties Questionnaire (SDQ): data from british parents, teachers and children. Journal of Abnormal Child Psychology.

[CR18] Goodman R (2001). Psychometric properties of the Strengths and Difficulties Questionnaire. Journal of the American Academy of Child & Adolescent Psychiatry.

[CR19] Hails KA, Reuben JD, Shaw DS, Dishion TJ, Wilson MN (2018). Transactional associations among maternal depression, parent–child coercion, and child conduct problems during early childhood. Journal of Clinical Child & Adolescent Psychology.

[CR20] Hammerton G, Zammit S, Mahedy L, Pearson RM, Sellers R, Thapar A, Collishaw S (2015). Pathways to suicide-related behavior in offspring of mothers with depression: the role of offspring psychopathology. Journal of the American Academy of Child & Adolescent Psychiatry.

[CR21] Jung T, Wickrama KA (2008). An introduction to latent class growth analysis and growth mixture modeling. Social and Personality Psychology Compass.

[CR22] Kapur N, Cooper J, O’Connor RC, Hawton K (2013). Non-suicidal self-injury v. attempted suicide: new diagnosis or false dichotomy?. The British Journal of Psychiatry.

[CR23] Kersten P, Czuba K, McPherson K, Dudley M, Elder H, Tauroa R, Vandal A (2016). A systematic review of evidence for the psychometric properties of the Strengths and Difficulties Questionnaire. International Journal of Behavioral Development.

[CR24] Kessler RC, Barker PR, Colpe LJ, Epstein JF, Gfroerer JC, Hiripi E, Howes MJ, Normand SLT, Manderscheid RW, Walters EE, Zaslavsky AM (2003). Screening for serious mental illness in the general population. Archives of General Psychiatry.

[CR25] Kuckertz JM, Mitchell C, Wiggins JL (2018). Parenting mediates the impact of maternal depression on child internalizing symptoms. Depression and Anxiety.

[CR26] Moretti MM, Obsuth I (2009). Effectiveness of an attachment-focused manualized intervention for parents of teens at risk for aggressive behaviour: the Connect Program. Journal of Adolescence.

[CR27] Murray AL, Eisner M, Nagin D, Ribeaud D (2020). A multi-trajectory analysis of commonly co-occurring mental health issues across childhood and adolescence. European Child & Adolescent Psychiatry.

[CR29] Murray AL, Nagin D, Obsuth I, Ribeaud D, Eisner M (2021). Young adulthood outcomes of joint mental health trajectories: a group-based trajectory model analysis of a 13-year longitudinal cohort study. Child Psychiatry & Human Development.

[CR31] Nock MK, Borges G, Bromet E, Cha CB, Kessler RC, Lee S (2008). Suicide and suicidal behavior. Epidemiologic Reviews.

[CR32] Odgers CL, Caspi A, Broadbent JM, Dickson N, Hancox RJ, Harrington H, Poulton R, Sears MR, Thomson WM, Moffitt TE (2007). Prediction of differential adult health burden by conduct problem subtypes in males. Archives of General Psychiatry.

[CR33] Orri M, Galera C, Turecki G, Forte A, Renaud J, Boivin M, Geoffroy MC (2018). Association of childhood irritability and depressive/anxious mood profiles with adolescent suicidal ideation and attempts. JAMA Psychiatry.

[CR34] Pilowsky DJ, Wickramaratne P, Talati A, Tang M, Hughes CW, Garber J, Weissman MM (2008). Children of depressed mothers 1 year after the initiation of maternal treatment: findings from the STAR* D-Child study. American Journal of Psychiatry.

[CR35] Patalay P, Moulton V, Goodman A, Ploubidis GB (2017). Cross-domain symptom development typologies and their antecedents: results from the UK Millennium Cohort Study. Journal of the American Academy of Child & Adolescent Psychiatry.

[CR36] Pineda J, Dadds MR (2013). Family intervention for adolescents with suicidal behavior: a randomized controlled trial and mediation analysis. Journal of the American Academy of Child & Adolescent Psychiatry.

[CR37] Rubin DB (1976). Inference and missing data. Biometrika.

[CR38] Russell AE, Heron J, Gunnell D, Ford T, Hemani G, Joinson C, Moran P, Relton C, Suderman M, Mars B (2019). Pathways between early-life adversity and adolescent self-harm: the mediating role of inflammation in the Avon Longitudinal Study of parents and children. Journal of Child Psychology and Psychiatry.

[CR39] Sameroff A, Mackenzie M (2003). Research strategies for capturing transactional models of development: the limits of the possible. Development and Psychopathology.

[CR40] Shaw DS, Sitnick SL, Reuben J, Dishion TJ, Wilson MN (2016). Transactional effects among maternal depression, neighborhood deprivation, and child conduct problems from early childhood through adolescence: a tale of two low-income samples. Development and Psychopathology.

[CR41] Sifaki M, Midouhas E, Papachristou E, Flouri E (2021). Reciprocal relationships between paternal psychological distress and child internalising and externalising difficulties from 3 to 14 years: a cross-lagged analysis. European Child & Adolescent Psychiatry.

[CR42] 10.1007/s00787-020-01642-0

[CR43] Skipstein A, Janson H, Kjeldsen A, Nilsen W, Mathiesen KS (2012). Trajectories of maternal symptoms of depression and anxiety over 13 years: the influence of stress, social support, and maternal temperament. Bmc Public Health.

[CR44] Speyer LG, Neaves S, Hall HA, Hemani G, Lombardo MV, Murray AL, Auyeung B, Luciano M (2021). Polygenic risks for joint developmental trajectories of internalizing and externalizing problems: findings from the ALSPAC cohort. Journal of Child Psychology and Psychiatry.

[CR45] Shi Q, Ettekal I (2021). Co-occurring trajectories of internalizing and externalizing problems from grades 1 to 12: longitudinal associations with teacher-child relationship quality and academic performance. Journal of Educational Psychology.

[CR46] van der Waerden J, Galéra C, Larroque B, Saurel-Cubizolles MJ, Sutter-Dallay AL, Melchior M (2015). Maternal depression trajectories and children’s behavior at age 5 years. The Journal of Pediatrics.

[CR47] Waraan L, Siqveland J, Hanssen-Bauer K, Czjakowski NO, Axelsdóttir B, Mehlum L, Aalberg M (2022). Family therapy for adolescents with depression and suicidal ideation: a systematic review and meta–analysis. Clinical Child Psychology and Psychiatry.

[CR48] World Health Organization (2021). *Suicide worldwide in 2019*. Retrieved 16 January 2022, from https://www.who.int/publications-detail-redirect/9789240026643

[CR49] Wickersham A, Leightley D, Archer M, Fear NT (2020). The association between paternal psychopathology and adolescent depression and anxiety: a systematic review. Journal of Adolescence.

