# Supplementary material for: Co-Developmental Trajectories of Parental Psychological Distress and Child Internalizing and Externalizing Problems in Childhood and Adolescence: Associations with Self-Harm and Suicide Attempts
Source: Res Child Adolesc Psychopathol. 2023 Feb 7;51(6):847–58. doi: 10.1007/s10802-023-01034-3 (PMC10195721; doi:10.1007/s10802-023-01034-3)
Supplement: Supplementary file 1 — Supplementary Material 1 [file 10802_2023_1034_MOESM1_ESM.docx]

**Sex Differences**

It is also important to consider the sex of children and parents. For example, it has been posited that parents exert the greatest influence on their same-sex offspring because, e.g., children are more strongly influenced by models that are more similar to themselves, or that it is easier for same-sex parent–child dyads to establish attachment relationships (e.g., Diener et al., 2008). Additionally, given that mothers typically take on more parenting responsibilities and spend more time with their children (e.g., Pew Research Center, 2013; Speight, 2011), mothers’ mental illness may have a stronger impact on children’s functioning than fathers’. Also, previous studies have shown that male and female youth are more susceptible to developing externalizing and internalizing problems, respectively (e.g., Murray et al., 2021; Speyer et al., 2021).

Prior empirical work has shown that maternal psychological distress (e.g., depression) may have a greater effect on a female offspring’s internalizing problems than on those of male offspring (e.g., Livings, 2021), while other studies have found no such sex differences (e.g., Middeldorp et al., 2016). Regarding externalizing problems, findings are also mixed, with research finding a transactional relation between externalizing problems and paternal and maternal distress in female offspring but not in male offspring (Fanti et al., 2013), a stronger father-son relation (Cummings et al., 2005), or no sex differences (e.g., Middeldorp et al., 2016). Thus, exploring sex differences in a nationally representative sample may help clarify these inconsistent previous findings.

**Sex-Stratified Analyses**

Given previous studies have shown sex and gender differences in the (joint) developmental trajectories of males and females (including differing numbers of trajectory groups in the optimal models), sex-stratified analyses were used to characterize the joint trajectories of male versus female youth (Murray et al., 2022). This method and the sample size available make it possible to detect different optimal models for males and females.

Online Supplementary Table S8 presents statistical fit indices for sex-stratified analyses. For females, the LMR-test indicated the four-class solution as optimal for both the linear growth model and the linear + quadratic growth model. The four-class linear + quadratic model was selected as the final model since it had lower AIC, BIC, and SaBIC values, compared to the four-class linear model. For the same reason, a 3-class linear and quadratic growth model was determined as the males’ optimal model. The four trajectories that emerged in the female sample were very similar to those that were identified in the whole sample and were thus given the same labels: “low symptoms” (59.8% of females), “moderate symptoms in children” (23.0%), “notable symptoms in fathers” (10.1%), and “co-occurring maternal and child symptoms” (7.1%). For males, the first class (64.3% of males) was comparable to the first class that emerged in the whole sample and was thus labeled “low symptoms”. The second class (25.9%) showed initially moderate and increasing child internalizing problems, initially relatively high and slightly decreasing child externalizing problems, initially moderate-low and slightly increasing maternal and paternal distress, and was, therefore, labeled “notable externalizing problems in children”. The third class (9.8%) showed initially high/high-moderate and slightly increasing maternal distress/child internalizing problems, initially relatively high and decreasing child externalizing problems, and initially moderate and increasing paternal distress. This class was, therefore, labeled “co-occurring maternal and child symptoms”. The final selected models in the female and male sample are summarized in online Supplementary Table S9 and plotted in Figures S1 and S2.

In females, the co-occurring maternal and child symptoms group showed the highest risk of self-harm at ages 14 and 17, and lifetime suicide attempts, followed by the group of moderate symptoms in children, notable symptoms in fathers, and low symptoms, with an exception: youth in the group of notable symptoms in fathers reported slightly higher self-harm at age 17 compared to the group of moderate symptoms in children. In males, co-occurring maternal and child symptoms group showed the highest risk of self-harm at ages 14 and 17, and lifetime suicide attempts, followed by the trajectory of notable externalizing problems in children, and low symptoms. However, some of these did not reach a statistically significant level; see online Supplementary Table S10 for details.

**Discussion Regarding Sex Differences**

The results of sex-stratified analyses showed that, in the female sample, the identified co-developmental patterns of parental psychological distress and child internalizing and externalizing problems were comparable to those in the whole sample. However, in the male sample, a group with notable externalizing problems (25.9%) was detected, and their internalizing problems were only moderate. This result was in line with the previous findings that externalizing problems are more prominent in male youth (e.g., Murray et al., 2022). Additionally, in the group of co-occurring maternal and child symptoms, externalizing problems in male youth were quite high, but their internalizing problems were not as high as those of the group with the same label in the female sample. This result might indicate that maternal distress is closely related to both internalizing and externalizing problems in female youth, whereas maternal distress is not as closely associated with internalizing problems in male youth as it is in female youth. It may, however, also reflect a broader sex difference in vulnerabilities rather than specifically in the context of maternal functioning. Further research will be needed to clarify this. There was also a group with notable paternal distress, but their daughters’ problem behaviors were at low or moderate levels. This group was not present in the male sample, possibly suggesting that female youth’s behavioral and emotional issues are more independent of their paternal psychological distress. Collectively, these aforementioned findings suggest that it is worthwhile to investigate whether male and female youth can benefit from tailored, two-generation interventions that place a greater emphasis on parental distress and externalizing issues in male children and on maternal distress and internalizing and externalizing issues in their female offspring. However, it should be noted that children’s biological sex was collected in the MCS study, and the results may not be applicable to gender fluid, questioning, non-binary, or transgender children.

**Additional Analyses**

Additional analyses were conducted to provide more detail on the current investigation. First, we also analyzed how demographic factors (parental ethnicity, education level, and economic activity status) were related to the trajectory class membership using the automatic 3-Step approach (Asparouhov & Muthén, 2014). The low symptoms group was employed as the reference group, and results showed that children from families with racially minoritized fathers, mothers and fathers without college degrees, unemployed mothers and fathers were more likely to be in the groups of moderate symptoms in children and notable symptoms in fathers, compared to the low symptoms group. The abovementioned factors, besides racially minoritized fathers, were also associated with membership in the co-occurring maternal and child symptoms group compared to the low symptoms group (online Supplementary Table S11).

Second, to account for stratification, clustering, and weighting, we included them in the final selection model (i.e., 4-class model). In the adjusted model, the shape of trajectories (which included data from ages 3-14, and it was thus estimated based on attrition weights at age 14) and their links to age 14 self-harm (estimates based on attrition weights at age 14), age 17 self-harm (estimates based on attrition weights at age 17) and lifetime suicide attempts (reported at age 17, and estimates based on attrition weights at age 17) outcomes were generally in line with unadjusted results (online Supplementary Figure S3 and Tables S12 and S13). Third, since genetics might play an important role in the link between parents and children’s mental health issues, analyses were also conducted in the sample with biological parents (not in the sample with non-biological parents because there was no comparable sample size to that of the biological parent sample. n =11560 biological parents, n =41 non-biological mothers, and n =933 non-biological fathers). Results indicated both 3-class and 4-class as optimal models in the biological parent sample based on the LMR test and the information statistics AIC, BIC, and saBIC (online Supplementary Table S14). The models are summarized in online Supplementary Table S15 and Figures S4 and S5. In the 3-class model, the group of notable symptoms in fathers found in the whole sample (both including children with biological and non-biological parents) was not detected in the biological parent sample. In the 4-class model, the identified joint trajectory groups were almost identical to the groups detected in the whole sample. Small differences are the levels of internalizing and externalizing problems in the co-occurring maternal and child symptoms group, which are generally slightly lower in the biological parent sample than those in the whole sample. Additionally, the associations between trajectories and self-harm at ages 14 and 17 and lifetime suicide attempts in the biological parent sample were generally consistent with those found in the whole sample (online Supplementary Table S16).

**References**

Asparouhov, T., & Muthén, B. (2014). Auxiliary variables in mixture modeling: Using the BCH method in Mplus to estimate a distal outcome model and an arbitrary secondary model. *Mplus Web Notes*, *21*(2), 1-22.

Cummings, E. M., Keller, P. S., & Davies, P. T. (2005). Towards a family process model of maternal and paternal depressive symptoms: Exploring multiple relations with child and family functioning. *Journal of Child Psychology and Psychiatry*, *46*(5), 479–489. https://doi.org/10.1111/j.1469-7610.2004.00368.x

Diener, M. L., Isabella, R. A., Behunin, M. G., & Wong, M. S. (2008). Attachment to mothers and fathers during middle childhood: Associations with child gender, grade, and competence. *Social Development*, *17*(1), 84-101. https://doi.org/10.1111/j.1467-9507.2007.00416.x

Livings, M. S. (2021). The gendered relationship between maternal depression and adolescent internalizing symptoms. *Social Science & Medicine*, *291*, 114464. https://doi.org/10.1016/j.socscimed.2021.114464

Middeldorp, C. M., Wesseldijk, L. W., Hudziak, J. J., Verhulst, F. C., Lindauer, R. J. L., & Dieleman, G. C. (2016). Parents of children with psychopathology: Psychiatric problems and the association with their child’s problems. *European Child & Adolescent Psychiatry*, *25*(8), 919–927. https://doi.org/10.1007/s00787-015-0813-2

Murray, A. L., Ushakova, A., Speyer, L., Brown, R., Auyeung, B., & Zhu, X. (2022). Sex/gender differences in individual and joint trajectories of common mental health symptoms in early to middle adolescence. *JCPP Advances*, *2*(1), e12057. https://doi.org/10.1002/jcv2.12057

Pew Research Center (2013). Chapter 4: How Mothers and Fathers Spend Their Time. *Pew Research Center's Social & Demographic Trends Project*. https://www.pewresearch.org/social-trends/2013/03/14/chapter-4-how-mothers-and-fathers-spend-their-time/

Speight, S. (2011). Gender Convergence in Domestic Work: Discerning the Effects of Interactional and Institutional Barriers from Large-scale Data. *Sociology*, *45*(2), 234–251. https://doi.org/10.1177/0038038510394014

Speyer, L. G., Neaves, S., Hall, H. A., Hemani, G., Lombardo, M. V., Murray, A. L., Auyeung, B., & Luciano, M. (2021). Polygenic risks for joint developmental trajectories of internalizing and externalizing problems: Findings from the ALSPAC cohort. *Journal of Child Psychology and Psychiatry*, *63*(8), 948-956. https://doi.org/10.1111/jcpp.13549

**Table S1: The number (percentage) of participants with valid data from at least 1, 2, 3, 4, and 5 waves (i.e., at ages 3, 5, 7, 11, and 14) on each construct and across families**

| Maternal distress | At least 1 wave | 12520 | 100.00% |
| --- | --- | --- | --- |
|  | At least 2 waves | 11581 | 92.50% |
|  | At least 3 waves | 10420 | 83.23% |
|  | At least 4 waves | 8917 | 71.22% |
|  | 5 waves | 6413 | 51.22% |
| Paternal distress | At least 1 wave | 12520 | 100.00% |
|  | At least 2 waves | 10181 | 81.32% |
|  | At least 3 waves | 8182 | 65.35% |
|  | At least 4 waves | 6211 | 49.61% |
|  | 5 waves | 3908 | 31.21% |
| Child internalizing | At least 1 wave | 12520 | 100.00% |
|  | At least 2 waves | 11897 | 95.02% |
|  | At least 3 waves | 10917 | 87.20% |
|  | At least 4 waves | 9463 | 75.58% |
|  | 5 waves | 6996 | 55.88% |
| Child externalizing | At least 1 wave | 12520 | 100.00% |
|  | At least 2 waves | 11897 | 95.02% |
|  | At least 3 waves | 10917 | 87.20% |
|  | At least 4 waves | 9464 | 75.59% |
|  | 5 waves | 6996 | 55.88% |
| Participating percentage across families | At least 1 wave | 12520 | 100.00% |
|  | At least 2 waves | 11931 | 95.30% |
|  | At least 3 waves | 11002 | 87.88% |
|  | At least 4 waves | 9640 | 77.00% |
|  | 5 waves | 7351 | 58.71% |

**Table S2:** **Maternal characteristics reported at sweep 1** (children aged 9 months, n =12520)

| **Maternal characteristics** |  | **n** | **Proportion** |
| --- | --- | --- | --- |
| **Biological status** | Biological | 12479 | 99.7% |
|  | Non-biological | 41 | .3% |
| **Maternal age at birth of child** | 12 to 19 year-old | 731 | 5.8% |
|  | 20 to 29 year-old | 5302 | 42.3% |
|  | 30 to 39 year-old | 5764 | 46.0% |
|  | 40 plus year-old | 276 | 2.2% |
|  | Missing | 447 | 3.6% |
| **Maternal age at interview** | 14 to 19 year-old | 457 | 3.7% |
|  | 20 to 29 year-old | 4875 | 38.9% |
|  | 30 to 39 year-old | 6299 | 50.3% |
|  | 40 plus year-old | 442 | 3.5% |
|  | Missing | 447 | 3.6% |
| **Maternal ethnicity** | White | 10542 | 84.2 |
|  | Mixed | 80 | .6% |
|  | Indian | 324 | 2.6% |
|  | Pakistani and Bangladeshi | 627 | 5.0% |
|  | Black or Black British | 265 | 2.1% |
|  | Other Ethnic group (inc Chinese, Other) | 203 | 1.6% |
|  | Missing | 479 | 3.8% |
| **Maternal education level** | Higher degree | 498 | 4.0% |
|  | First degree | 1896 | 15.1% |
|  | Diplomas in higher education | 1186 | 9.5% |
|  | A/AS/S levels | 1271 | 10.2% |
|  | O level/GCSE grades A-C | 4032 | 32.2% |
|  | GCSE grades D-G | 1173 | 9.4% |
|  | Other academic qualifications | 316 | 2.5% |
|  | None of these qualifications | 1673 | 13.4% |
|  | Missing | 475 | 3.8% |
| **Maternal economic activity status** | Employed | 6074 | 48.5% |
|  | Self employed | 470 | 3.8% |
|  | Looking for work | 1 | .0% |
|  | Poor health | 1 | .0% |
|  | Non-working for other/unknown reason | 5503 | 44.0% |
|  | Missing | 471 | 3.8% |

**Table S3:** **Paternal characteristics reported at sweep 1** (children aged 9 months, n =12520)

| **Paternal characteristics** |  | **n** | **Proportion** |
| --- | --- | --- | --- |
| **Biological status** | Biological | 11587 | 92.5% |
|  | Non-biological | 933 | 7.5% |
| **Paternal age at birth of child** | 12 to 19 year-old | 103 | .8% |
|  | 20 to 29 year-old | 3265 | 26.1% |
|  | 30 to 39 year-old | 6247 | 49.9% |
|  | 40 plus year-old | 1093 | 8.7% |
|  | Missing | 1812 | 14.5% |
| **Paternal age at interview** | 14 to 19 year-old | 54 | .4% |
|  | 20 to 29 year-old | 2752 | 22.0% |
|  | 30 to 39 year-old | 6546 | 52.3% |
|  | 40 plus year-old | 1356 | 10.8% |
|  | Missing | 1812 | 14.5% |
| **Paternal ethnicity** | White | 8516 | 68.0% |
|  | Mixed | 63 | .5% |
|  | Indian | 270 | 2.2% |
|  | Pakistani and Bangladeshi | 478 | 3.8% |
|  | Black or Black British | 196 | 1.6% |
|  | Other Ethnic group (inc Chinese, Other) | 164 | 1.3% |
|  | Missing | 2833 | 22.6% |
| **Paternal education level** | Higher degree | 617 | 4.9% |
|  | First degree | 1583 | 12.6% |
|  | Diplomas in higher education | 891 | 7.1% |
|  | A/AS/S levels | 791 | 6.3% |
|  | O level/GCSE grades A-C | 3053 | 24.4% |
|  | GCSE grades D-G | 911 | 7.3% |
|  | Other academic qualifications | 238 | 1.9% |
|  | None of these qualifications | 1598 | 12.8% |
|  | Missing | 2838 | 22.7% |
| **Paternal economic activity status** | Employed | 7295 | 58.3% |
|  | Self employed | 1500 | 12.0% |
|  | Looking for work | 430 | 3.4% |
|  | Poor health | 222 | 1.8% |
|  | Non-working for other/unknown reason | 42 | .3% |
|  | Missing | 57 | .5% |

**Table S4: Descriptive statistics**

| Construct | *n* | *M* | *SD* | Minimum | Maximum | Construct | *n* | *M* | *SD* | Minimum | Maximum |
| --- | --- | --- | --- | --- | --- | --- | --- | --- | --- | --- | --- |
| Age3 MPD | 10592 | 3.13 | 3.61 | 0 | 24 | Age 3 IN | 11236 | 2.81 | 2.48 | 0 | 20 |
| Age5 MPD | 11013 | 2.99 | 3.66 | 0 | 24 | Age 5 IN | 11305 | 2.42 | 2.46 | 0 | 18 |
| Age7 MPD | 10207 | 2.95 | 3.68 | 0 | 24 | Age 7 IN | 10457 | 2.60 | 2.70 | 0 | 20 |
| Age11 MPD | 9617 | 3.80 | 4.20 | 0 | 24 | Age 11 IN | 9977 | 3.06 | 3.04 | 0 | 19 |
| Age14 MPD | 8422 | 4.17 | 4.08 | 0 | 24 | Age 14 IN | 8818 | 3.58 | 3.33 | 0 | 19 |
| Age3 FPD | 9125 | 2.91 | 3.19 | 0 | 24 | Age 3 EX | 11236 | 6.46 | 3.71 | 0 | 20 |
| Age5 FPD | 9372 | 2.95 | 3.36 | 0 | 24 | Age 5 EX | 11305 | 4.56 | 3.30 | 0 | 20 |
| Age7 FPD | 8314 | 2.98 | 3.49 | 0 | 24 | Age 7 EX | 10457 | 4.51 | 3.47 | 0 | 20 |
| Age11 FPD | 7794 | 3.84 | 3.92 | 0 | 24 | Age 11 EX | 9977 | 4.25 | 3.46 | 0 | 20 |
| Age14 FPD | 6397 | 3.64 | 3.63 | 0 | 24 | Age 14 EX | 8819 | 4.17 | 3.45 | 0 | 20 |
|  |  | n (participants with self-harm or suicide attempts) | | | |  |  |  |  |  |  |
| Age 14 SH | 8757 | 1256 |  |  |  |  |  |  |  |  |  |
| Age 17 SH | 7685 | 1764 |  |  |  |  |  |  |  |  |  |
| Lifetime SA | 7673 | 529 |  |  |  |  |  |  |  |  |  |

*Note.* MPD=Maternal psychological distress, FPD=Paternal psychological distress, IN=Internalizing problems, EX=Externalizing problems, SH=Self-harm, SA= Suicide attempts.

**Table S5: Pearson correlations between main study variables**

|  | 1 | 2 | 3 | 4 | 5 | 6 | 7 | 8 | 9 | 10 |
| --- | --- | --- | --- | --- | --- | --- | --- | --- | --- | --- |
| 1. Age 3 IN | _ |  |  |  |  |  |  |  |  |  |
| 2. Age 5 IN | .492*** | _ |  |  |  |  |  |  |  |  |
| 3. Age 7 IN | .415*** | .578*** | _ |  |  |  |  |  |  |  |
| 4. Age 11 IN | .331*** | .446*** | .557*** | _ |  |  |  |  |  |  |
| 5. Age 14 IN | .298*** | .382*** | .460*** | .612*** | _ |  |  |  |  |  |
| 6. Age 3 EX | .368*** | .289*** | .297*** | .282*** | .268*** | _ |  |  |  |  |
| 7. Age 5 EX | .263*** | .378*** | .345*** | .325*** | .292*** | .597*** | _ |  |  |  |
| 8. Age 7 EX | .237*** | .301*** | .438*** | .368*** | .320******* | .536*** | .702*** | _ |  |  |
| 9. Age 11 EX | .215*** | .266*** | .339*** | .473*** | .376*** | .478*** | .603*** | .700*** | _ |  |
| 10. Age 14 EX | .199*** | .238*** | .296*** | .355*** | .447*** | .428*** | .529*** | .601*** | .711*** | _ |
| 11. Age3 MPD | .275*** | .241*** | .250*** | .251*** | .225*** | .286*** | .236*** | .204*** | .215*** | .197*** |
| 12. Age5 MPD | .225*** | .307*** | .286*** | .256*** | .234*** | .227*** | .286*** | .245*** | .236*** | .204*** |
| 13. Age7 MPD | .219*** | .246*** | .336*** | .275*** | .233*** | .221*** | .254*** | .278*** | .255*** | .234*** |
| 14. Age11 MPD | .227*** | .255*** | .289*** | .355*** | .285*** | .224*** | .234*** | .244*** | .317*** | .269*** |
| 15. Age14 MPD | .205*** | .237*** | .261*** | .303*** | .317*** | .224*** | .224*** | .215*** | .263*** | .272*** |
| 16. Age3 FPD | .115*** | .109*** | .112*** | .117*** | .115*** | .108*** | .105*** | .113*** | .087*** | .103*** |
| 17. Age5 FPD | .093*** | .107*** | .106*** | .105*** | .111*** | .117*** | .121*** | .119*** | .098*** | .092*** |
| 18. Age7 FPD | .094*** | .107*** | .132*** | .126*** | .130*** | .111*** | .115*** | .118*** | .101*** | .107*** |
| 19. Age11 FPD | .105*** | .105*** | .129*** | .163*** | .150*** | .109*** | .106*** | .125*** | .149*** | .152*** |
| 20. Age14 FPD | .098*** | .113*** | .116*** | .142*** | .167*** | .082*** | .104*** | .101*** | .130*** | .162*** |
| 21. Age 14 SH | .002 | .024* | .038** | .084*** | .171*** | .026* | .032** | .037** | .047*** | .097*** |
| 22. Age 17 SH | .002 | .012 | .034** | .077*** | .135*** | .018 | .017 | .021 | .044*** | .051*** |
| 23. Lifetime SA | .016 | .026* | .045*** | .097*** | .163*** | .053*** | .078*** | .059*** | .075*** | .111*** |

*Note.* MPD=Maternal psychological distress, FPD=Paternal psychological distress, IN=Internalizing problems, EX=Externalizing problems, SH=Self-harm, SA= Suicide attempts. **p* < .05; ***p* < .01; ****p* < .001

**Table S5 (continued)**

|  | 11 | 12 | 13 | 14 | 15 | 16 | 17 | 18 | 19 | 20 | 21 | 22 |
| --- | --- | --- | --- | --- | --- | --- | --- | --- | --- | --- | --- | --- |
| 11. Age3 MPD | _ |  |  |  |  |  |  |  |  |  |  |  |
| 12. Age5 MPD | .565*** | _ |  |  |  |  |  |  |  |  |  |  |
| 13. Age7 MPD | .535*** | .597*** | _ |  |  |  |  |  |  |  |  |  |
| 14. Age11 MPD | .481*** | .514*** | .557*** | _ |  |  |  |  |  |  |  |  |
| 15. Age14 MPD | .468*** | .486*** | .510*** | .610*** | _ |  |  |  |  |  |  |  |
| 16. Age3 FPD | .181*** | .130*** | .126*** | .143*** | .123*** | _ |  |  |  |  |  |  |
| 17. Age5 FPD | .146*** | .186*** | .147*** | .132*** | .136*** | .554*** | _ |  |  |  |  |  |
| 18. Age7 FPD | .139*** | .172*** | .194*** | .168*** | .138*** | .521*** | .586*** | _ |  |  |  |  |
| 19. Age11 FPD | .132*** | .149*** | .156*** | .219*** | .148*** | .445*** | .505*** | .555*** | _ |  |  |  |
| 20. Age14 FPD | .135*** | .155*** | .113*** | .176*** | .190*** | .451*** | .488*** | .537*** | .616*** | _ |  |  |
| 21. Age 14 SH | .046*** | .044*** | .035** | .063*** | .085*** | .005 | .015 | .015 | .039** | .048*** | _ |  |
| 22. Age 17 SH | .054*** | .042*** | .041** | .054*** | .083*** | .039** | .054*** | .026* | .011 | .043** | .300*** | _ |
| 23. Lifetime SA | .074*** | .079*** | .079*** | .095*** | .127*** | .037** | .041** | .072*** | .043** | .048*** | .312*** | .362*** |

*Note.* MPD=Maternal psychological distress, FPD=Paternal psychological distress, IN=Internalizing problems, EX=Externalizing problems, SH=Self-harm, SA= Suicide attempts. **p*< .05; ***p*< .01; ****p*< .001

**Table S6: Model fits for the 1-8 class models in the whole sample**

| Model | LMR | *p* | AIC | BIC | saBIC | Entropy |  | Model | LMR | *P* | AIC | BIC | saBIC | Entropy |  |
| --- | --- | --- | --- | --- | --- | --- | --- | --- | --- | --- | --- | --- | --- | --- | --- |
| **Model with linear and quadratic growth** | | | | | | |  | **Model with linear growth** | | | | | | | |
| 1-class | - | - | 1022611.155 | 1022849.078 | 1022747.385 | N/A |  | 1-class | - | - | 1023859.069 | 1024067.251 | 1023978.270 | N/A |  |
| 2-class | 34839.116 | <.001 | 987514.000 | 987848.579 | 987705.573 | .886 |  | 2-class | 34241.713 | <.001 | 989232.113 | 989507.211 | 989389.628 | .884 |  |
| 3-class | 9455.796 | .083 | 978007.112 | 978438.347 | 978254.029 | .872 |  | 3-class | 9284.935 | <.001 | 979855.835 | 980197.849 | 980051.666 | .870 |  |
| **4-class** | **7425.795** | **.036** | **970546.775** | **971074.666** | **970849.035** | **.870** |  | 4-class | 7268.901 | .097 | 972519.332 | 972928.262 | 972753.478 | .871 |  |
| 5-class | 5691.475 | .096 | 964834.898 | 965459.445 | 965192.501 | .879 |  | 5-class | 5526.396 | .087 | 966945.856 | 967421.701 | 967218.316 | .878 |  |
| 6-class | 4242.879 | .567 | 960583.427 | 961304.630 | 960996.375 | .850 |  | 6-class | 4082.052 | .056 | 962833.732 | 963376.493 | 963144.507 | .847 |  |
| 7-class | 3165.960 | .175 | 957417.656 | 958235.515 | 957885.947 | .854 |  | 7-class | 3107.520 | .006 | 959707.617 | 960317.294 | 960056.706 | .851 |  |
| 8-class | 3024.775 | .002 | 954394.220 | 955308.735 | 954917.854 | .852 |  | 8-class | 2947.040 | .030 | 956743.871 | 957420.464 | 957131.275 | .848 |  |

*Note.* Solution(s) considered “best-fitting” indicated in bold.

**Table S7: Growth parameters for the selected 4-class model in the whole sample**

| Class Label (class size*) | Domain | Maternal distress | | | Paternal distress | | | Internalizing problems | | | Externalizing problems | | |
| --- | --- | --- | --- | --- | --- | --- | --- | --- | --- | --- | --- | --- | --- |
|  | Parameter | Intercept | Linear | Quadratic | Intercept | Linear | Quadratic | Intercept | Linear | Quadratic | Intercept | Linear | Quadratic |
| Low symptoms (59.0%) | Estimate | 2.01 | -1.28 | 2.21 | 2.04 | .03 | .81 | 2.00 | -2.15 | 2.57 | 4.61 | -6.85 | 5.10 |
|  | SE | .04 | .13 | .13 | .04 | .14 | .14 | .03 | .10 | .10 | .08 | .13 | .12 |
| Moderate symptoms in children (22.5%) | Estimate | 3.37 | -.53 | 2.38 | 2.48 | -.03 | .97 | 3.63 | .09 | 1.79 | 8.67 | -4.32 | 2.81 |
|  | SE | .11 | .38 | .34 | .08 | .32 | .30 | .12 | .43 | .32 | .21 | .47 | .38 |
| Notable symptoms in fathers (10.7%) | Estimate | 4.02 | -.41 | 2.23 | 7.41 | 7.22 | -5.30 | 3.05 | -2.12 | 3.18 | 6.39 | -7.54 | 5.69 |
|  | SE | .31 | .57 | .55 | .26 | .97 | .92 | .11 | .34 | .34 | .16 | .43 | .38 |
| Co-occurring maternal and child symptoms (7.8%) | Estimate | 10.08 | 2.12 | -.95 | 4.20 | 2.82 | -.89 | 5.40 | 3.24 | -1.06 | 9.60 | -4.29 | 2.76 |
|  | SE | .58 | 1.21 | 1.02 | .23 | .99 | .98 | .22 | 1.12 | .88 | .39 | .91 | .73 |

*Note*. *Based on estimated posterior probabilities.

**Table S8: Model fits for the 1-8 class models in the female (n =6145) and male (n =6375) sample**

| Model | LMR | *p* | AIC | BIC | saBIC | Entropy |  | Model | LMR | *p* | AIC | BIC | saBIC | Entropy |  |
| --- | --- | --- | --- | --- | --- | --- | --- | --- | --- | --- | --- | --- | --- | --- | --- |
| **Female: Model with linear and quadratic growth** | | | | | | |  | **Female: Model with linear growth** | | | | | | | |
| 1-class | - | - | 499891.172 | 500106.321 | 500004.633 | N/A |  | 1-class | - | - | 500770.870 | 500959.125 | 500870.148 | N/A |  |
| 2-class | 16537.388 | <.001 | 483233.957 | 483536.510 | 483393.512 | .882 |  | 2-class | 16198.667 | <.001 | 484383.878 | 484632.643 | 484515.067 | .879 |  |
| 3-class | 4536.888 | <.001 | 478683.062 | 479073.019 | 478888.711 | .863 |  | 3-class | 4450.658 | <.001 | 479894.531 | 480203.807 | 480057.631 | .861 |  |
| **4-class** | **3879.503** | **.015** | **474795.350** | **475272.711** | **475047.092** | **.874** |  | 4-class | 3826.854 | .003 | 476036.933 | 476406.720 | 476231.945 | .873 |  |
| 5-class | 2481.718 | .077 | 472317.748 | 472882.513 | 472615.584 | .846 |  | 5-class | 2372.300 | .234 | 473652.417 | 474082.714 | 473879.340 | .843 |  |
| 6-class | 2076.772 | .316 | 470248.663 | 470900.832 | 470592.593 | .848 |  | 6-class | 2000.681 | .148 | 471644.253 | 472135.061 | 471903.086 | .846 |  |
| 7-class | 1471.686 | .217 | 468789.999 | 469529.573 | 469180.022 | .856 |  | 7-class | 1418.712 | .110 | 470225.471 | 470776.789 | 470516.216 | .853 |  |
| 8-class | 1333.245 | .093 | 467470.997 | 468297.975 | 467907.114 | .844 |  | 8-class | 1277.077 | .094 | 468950.128 | 469561.956 | 469272.783 | .841 |  |
| **Male: Model with linear and quadratic growth** | | | | | | |  | **Male: Model with linear growth** | | | | | | | |
| 1-class | - | - | 521015.861 | 521232.185 | 521130.498 | N/A |  | 1-class | - | - | 521446.698 | 521635.982 | 521547.005 | N/A |  |
| 2-class | 18321.679 | <.001 | 502559.299 | 502863.505 | 502720.507 | .886 |  | 2-class | 18042.757 | <.001 | 503193.091 | 503443.216 | 503325.640 | .885 |  |
| **3-class** | **4940.769** | **.032** | **497601.145** | **497993.233** | **497808.924** | **.874** |  | 3-class | 4858.544 | .013 | 498290.923 | 498601.890 | 498455.713 | .873 |  |
| 4-class | 3576.906 | .167 | 494018.830 | 494498.800 | 494273.180 | .869 |  | 4-class | 3458.185 | .171 | 494806.875 | 495178.683 | 495003.907 | .869 |  |
| 5-class | 3230.627 | .090 | 490785.835 | 491353.686 | 491086.756 | .882 |  | 5-class | 3137.960 | .094 | 491647.115 | 492079.764 | 491876.388 | .881 |  |
| 6-class | 2198.806 | .201 | 488593.721 | 489249.454 | 488941.213 | .852 |  | 6-class | 2134.196 | .071 | 489503.849 | 489997.339 | 489765.364 | .849 |  |
| 7-class | 1733.115 | .013 | 486871.388 | 487615.003 | 487265.452 | .845 |  | 7-class | 1703.193 | .002 | 487797.054 | 488351.385 | 488090.810 | .842 |  |
| 8-class | 1653.181 | .382 | 485229.690 | 486061.188 | 485670.325 | .851 |  | 8-class | 1618.659 | .034 | 486175.864 | 486791.036 | 486501.862 | .849 |  |

*Note.* Solution(s) considered “best-fitting” indicated in bold.

**Table S9: Growth parameters for the selected model in the female and male sample**

| Class Label (class size*) | Domain | Maternal distress | | | Paternal distress | | | Internalizing problems | | | Externalizing problems | | | |
| --- | --- | --- | --- | --- | --- | --- | --- | --- | --- | --- | --- | --- | --- | --- |
| **Females** | Parameter | Intercept | Linear | Quadratic | Intercept | Linear | Quadratic | Intercept | Linear | Quadratic | Intercept | Linear | Quadratic |  |
| Low symptoms (59.8%) | Estimate | 2.02 | -1.39 | 2.34 | 2.00 | -.04 | .84 | 1.91 | -2.06 | 2.77 | 4.28 | -6.95 | 5.24 |  |
|  | SE | .05 | .18 | .19 | .05 | .19 | .19 | .04 | .14 | .14 | .10 | .17 | .16 |  |
| Moderate symptoms in children (23.0%) | Estimate | 3.36 | -.43 | 2.79 | 2.57 | -.07 | 1.11 | 3.66 | .15 | 2.11 | 8.08 | -5.94 | 4.15 |  |
|  | SE | .15 | .53 | .49 | .11 | .47 | .43 | .18 | .51 | .41 | .23 | .52 | .45 |  |
| Notable symptoms in fathers (10.1%) | Estimate | 3.93 | -1.28 | 3.21 | 7.78 | 6.08 | -4.22 | 3.01 | -2.31 | 3.48 | 6.00 | -8.88 | 6.79 |  |
|  | SE | .34 | .81 | .79 | .37 | 1.46 | 1.36 | .16 | .51 | .50 | .22 | .65 | .58 |  |
| Co-occurring maternal and child symptoms (7.1%) | Estimate | 10.33 | 2.24 | -1.15 | 4.12 | 3.11 | -1.23 | 5.32 | .53 | 1.86 | 9.08 | -5.21 | 3.45 |  |
|  | SE | .73 | 1.62 | 1.48 | .34 | 1.57 | 1.65 | .25 | 1.06 | .91 | .35 | 1.11 | .91 |  |
| **Males** |  |  |  |  |  |  |  |  |  |  |  |  |  |  |
| Low symptoms (64.3 %) | Estimate | 2.07 | -1.13 | 2.06 | 2.43 | .56 | .28 | 2.12 | -2.22 | 2.31 | 5.06 | -6.56 | 4.79 |  |
|  | SE | .05 | .18 | .18 | .06 | .21 | .21 | .04 | .14 | .13 | .09 | .18 | .17 |  |
| Notable externalizing problems in children (25.9%) | Estimate | 3.46 | -.16 | 1.71 | 3.49 | 1.42 | -.43 | 3.73 | .41 | 1.44 | 9.09 | -3.00 | 1.85 |  |
|  | SE | .14 | .47 | .44 | .19 | .50 | .48 | .18 | .49 | .39 | .22 | .55 | .46 |  |
| Co-occurring maternal and child symptoms (9.8%) | Estimate | 10.18 | 1.22 | .18 | 4.59 | 2.89 | -.70 | 5.00 | 2.79 | -1.38 | 9.18 | -4.95 | 3.35 |  |
|  | SE | .44 | 1.43 | 1.29 | .37 | 1.24 | 1.18 | .35 | .99 | .83 | .38 | .95 | .85 |  |

*Note*. *Based on estimated posterior probabilities.

**Table S10: Relations of the identified trajectory groups to outcomes of self-harm and suicide attempts in the female and male sample**

|  | Outcome means (SE) by class | | | | | | Wald test *p* value | | | | | |
| --- | --- | --- | --- | --- | --- | --- | --- | --- | --- | --- | --- | --- |
| **Females** | Low symptoms (c1) | Moderate symptoms in children (c2) | | Notable symptoms in fathers (c3) | | Co-occurring maternal and child symptoms (c4) | c1 vs. c2 | c1 vs. c3 | c1 vs. c4 | c2 vs. c3 | c2 vs. c4 | c3 vs. c4 |
| Age14 self-harm | .19 (.01) ^2,4^ | .24 (.02) ^1,4^ | | .19 (.02) ^4^ | | .33 (.03) ^1,2,3^ | .006** | .781 | <.001*** | .106 | .007** | <.001*** |
| Age17 self-harm | .26 (.02) ^2,3,4^ | .32 (.02) ^1^ | | .33 (.02) ^1^ | | .37 (.02) ^1^ | .006** | .028* | .002** | .888 | .202 | .300 |
| Lifetime suicide attempts (reported at age 17) | .07 (.01) ^2,3,4^ | .13 (.01) ^1,4^ | | .11 (.02) ^1,4^ | | .23 (.03) ^1,2,3^ | <.001*** | .042* | <.001*** | .356 | .002** | .001** |
| **Males** | Low symptoms (c1) | | Notable externalizing problems in children (c2) | | Co-occurring maternal and child symptoms (c3) | | c1 vs. c2 | c1 vs. c3 | c2 vs. c3 |  |  |  |
| Age14 self-harm | .06 (.01) ^2,3^ | | .12 (.01) ^1^ | | .09 (.02) ^1^ | | <.001*** | .048* | .239 |  |  |  |
| Age17 self-harm | .15 (.01) ^2^ | | .19 (.02) ^1^ | | .22 (.02) | | <.001*** | .065† | .403 |  |  |  |
| Lifetime suicide attempts (reported at age 17) | .03 (.00) ^2,3^ | | .07 (.01) ^1^ | | .07 (.02) ^1^ | | <.001*** | .004** | .753 |  |  |  |

*Note.* Numbers in superscript refers to significantly different subgroups in the outcomes. P values indicate for the Wald tests.

**Table S11: Logistic regression of ethnicity and SES-related factors on trajectory class membership**

|  | OR | SE | *p* |
| --- | --- | --- | --- |
| **Moderate symptoms in children** |  |  |  |
| Maternal ethnicity (white vs. racially minoritized) | 0.91 | 0.18 | 0.579 |
| Paternal ethnicity (white vs. racially minoritized) | 1.63 | 0.18 | 0.005** |
| Maternal economic status (employed vs. unemployed) | 1.19 | 0.07 | 0.009** |
| Paternal economic status (employed vs. unemployed) | 1.77 | 0.11 | <.001*** |
| Maternal education level (with vs. without college degrees) | 1.79 | 0.08 | <.001*** |
| Paternal education level (with vs. without college degrees) | 1.70 | 0.08 | <.001*** |
| **Notable symptoms in fathers** |  |  |  |
| Maternal ethnicity (white vs. racially minoritized) | 1.24 | 0.22 | 0.322 |
| Paternal ethnicity (white vs. racially minoritized) | 2.00 | 0.22 | 0.001** |
| Maternal economic status (employed vs. unemployed) | 1.53 | 0.08 | <.001*** |
| Paternal economic status (employed vs. unemployed) | 3.55 | 0.12 | <.001*** |
| Maternal education level (with vs. without college degrees) | 1.28 | 0.10 | 0.015* |
| Paternal education level (with vs. without college degrees) | 1.54 | 0.10 | <.001*** |
| **Co-occurring maternal and child symptoms group** |  |  |  |
| Maternal ethnicity (white vs. racially minoritized) | 1.76 | 0.31 | 0.064 |
| Paternal ethnicity (white vs. racially minoritized) | 1.64 | 0.31 | 0.105 |
| Maternal economic status (employed vs. unemployed) | 2.47 | 0.11 | <.001*** |
| Paternal economic status (employed vs. unemployed) | 3.79 | 0.13 | <.001*** |
| Maternal education level (with vs. without college degrees) | 2.11 | 0.15 | <.001*** |
| Paternal education level (with vs. without college degrees) | 2.20 | 0.14 | <.001*** |

**Table S12: Growth parameters for the selected 4-class model in the whole sample (adjust stratification, clustering, and weighting)**

| Class Label (class size*) | Domain | Maternal distress | | | Paternal distress | | | Internalizing problems | | | Externalizing problems | | |
| --- | --- | --- | --- | --- | --- | --- | --- | --- | --- | --- | --- | --- | --- |
|  | Parameter | Intercept | Linear | Quadratic | Intercept | Linear | Quadratic | Intercept | Linear | Quadratic | Intercept | Linear | Quadratic |
| Low symptoms (56.9%) | Estimate | 2.06 | -1.35 | 2.31 | 2.14 | 0.22 | 0.54 | 2.06 | -2.18 | 2.59 | 4.79 | -7.25 | 5.42 |
|  | SE | 0.06 | 0.18 | 0.19 | 0.07 | 0.19 | 0.18 | 0.04 | 0.15 | 0.15 | 0.09 | 0.17 | 0.16 |
| Moderate symptoms in children (22.9%) | Estimate | 3.29 | -0.83 | 2.76 | 2.48 | -0.31 | 1.20 | 3.55 | 0.33 | 1.78 | 8.79 | -4.27 | 3.10 |
|  | SE | 0.17 | 0.49 | 0.48 | 0.14 | 0.43 | 0.38 | 0.15 | 0.50 | 0.42 | 0.23 | 0.57 | 0.46 |
| Notable symptoms in fathers (12.1%) | Estimate | 4.84 | -0.64 | 2.39 | 6.95 | 7.41 | -4.96 | 3.24 | -2.30 | 3.32 | 6.51 | -7.85 | 5.93 |
|  | SE | 0.50 | 0.80 | 0.77 | 0.39 | 1.37 | 1.23 | 0.21 | 0.50 | 0.50 | 0.19 | 0.55 | 0.50 |
| Co-occurring maternal and child symptoms (8.1%) | Estimate | 8.88 | 5.92 | -3.46 | 3.69 | 3.93 | -1.64 | 5.33 | 4.83 | -1.92 | 10.00 | -2.56 | 1.74 |
|  | SE | 0.63 | 1.56 | 1.44 | 0.33 | 1.56 | 1.55 | 0.25 | 1.16 | 1.00 | 0.46 | 1.13 | 1.00 |

*Note*. *Based on estimated posterior probabilities. It should be noted that the analyses of the trajectory model was based on attrition weights at ages 14.

**Table S13: Relations of the four trajectories to outcomes of self-harm and suicide attempts in the whole sample (adjust stratification, clustering, and weighting)**

|  | Outcome means (SE) by class | | | | Wald test *p* value | | | | | |
| --- | --- | --- | --- | --- | --- | --- | --- | --- | --- | --- |
|  | Low symptoms (c1) | Moderate symptoms in children (c2) | Notable symptoms in fathers (c3) | Co-occurring maternal and child symptoms (c4) | c1 vs. c2 | c1 vs. c3 | c1 vs. c4 | c2 vs. c3 | c2 vs. c4 | c3 vs. c4 |
| Age14 self-harm | .13 (.01) ^2,4^ | .18 (.01) ^1,3,4^ | .14 (.01) ^2,4^ | .27 (.03) ^1,2,3^ | .002** | .552 | <.001*** | .040* | .005** | <.001*** |
| Age17 self-harm | .20 (.02) ^2,3,4^ | .26 (.02) ^1^ | .27 (.02) ^1^ | .28 (.02) ^1^ | .007** | .007** | .010* | .875 | .735 | .828 |
| Lifetime suicide attempts (reported at age 17) | .05 (.01) ^2,3,4^ | .08 (.01) ^1,4^ | .09 (.01) ^1,4^ | .15 (.02) ^1,2,3^ | .010* | .004** | <.001*** | .325 | .004** | .031* |

*Note.* It should be noted that the analyses of the associations between trajectories and age 14 and age 17 outcomes were based on attrition weights at ages 14 and 17, respectively. Numbers in superscript refers to significantly different subgroups in the outcomes. P values indicate for the Wald tests.

**Table S14: Model fits for the 1-8 class models in the sample with biological parent**

| Model | LMR | *p* | AIC | BIC | saBIC | Entropy |  | Model | LMR | *p* | AIC | BIC | saBIC | Entropy |  |
| --- | --- | --- | --- | --- | --- | --- | --- | --- | --- | --- | --- | --- | --- | --- | --- |
| **Model with linear and quadratic growth** | | | | | | |  | **Model with linear growth** | | | | | | | |
| 1-class | - | - | 945141.437 | 945376.807 | 945275.115 | N/A |  | 1-class | - | - | 946322.471 | 946528.419 | 946439.439 | N/A |  |
| 2-class | 31199.281 | <.001 | 913711.623 | 914042.612 | 913899.607 | .882 |  | 2-class | 30663.124 | <.001 | 915313.167 | 915585.314 | 915467.732 | .880 |  |
| **3-class** | **8898.198** | **.024** | **904766.260** | **905192.868** | **905008.551** | **.869** |  | 3-class | 8736.778 | <.001 | 906490.625 | 906828.969 | 906682.786 | .868 |  |
| **4-class** | **6976.196** | **.059** | **897758.704** | **898280.930** | **898055.301** | **.871** |  | 4-class | 6850.739 | .002 | 899576.521 | 899981.063 | 899806.279 | .871 |  |
| 5-class | 5298.981 | .190 | 892442.152 | 893059.997 | 892793.055 | .877 |  | 5-class | 5110.292 | .411 | 894423.535 | 894894.275 | 894690.890 | .877 |  |
| 6-class | 3882.624 | .079 | 888553.603 | 889267.068 | 888958.814 | .849 |  | 6-class | 3708.013 | .104 | 890689.483 | 891226.420 | 890994.435 | .846 |  |
| 7-class | 2966.108 | .011 | 885589.107 | 886398.191 | 886048.624 | .855 |  | 7-class | 2917.652 | .005 | 887755.178 | 888358.313 | 888097.727 | .852 |  |
| 8-class | 2850.630 | .006 | 882741.038 | 883645.740 | 883254.861 | .854 |  | 8-class | 2900.143 | .094 | 884982.775 | 885652.108 | 885362.920 | .848 |  |

*Note.* Solution(s) considered “best-fitting” indicated in bold.

**Table S15: Growth parameters for the selected 3/4-class model in the sample with biological parents**

| Class Label (class size*) | Domain | Maternal distress | | | Paternal distress | | | Internalizing problems | | | Externalizing problems | | |
| --- | --- | --- | --- | --- | --- | --- | --- | --- | --- | --- | --- | --- | --- |
|  | Parameter | Intercept | Linear | Quadratic | Intercept | Linear | Quadratic | Intercept | Linear | Quadratic | Intercept | Linear | Quadratic |
| **3-class model** |  |  |  |  |  |  |  |  |  |  |  |  |  |
| Low symptoms (65.2 %) | Estimate | 2.03 | -1.21 | 2.20 | 2.42 | 0.41 | 0.41 | 2.03 | -2.14 | 2.55 | 4.61 | -6.84 | 5.08 |
|  | SE | 0.04 | 0.13 | 0.14 | 0.04 | 0.15 | 0.15 | 0.04 | 0.10 | 0.10 | 0.08 | 0.13 | 0.12 |
| Moderate symptoms in children (25.3 %) | Estimate | 3.26 | -0.40 | 2.29 | 3.33 | 0.97 | 0.05 | 3.65 | 0.01 | 1.89 | 8.58 | -4.64 | 3.00 |
|  | SE | 0.10 | 0.40 | 0.37 | 0.18 | 0.42 | 0.39 | 0.15 | 0.40 | 0.30 | 0.23 | 0.45 | 0.38 |
| Co-occurring maternal and child symptoms (9.5 %) | Estimate | 9.73 | 2.04 | -0.63 | 4.82 | 3.72 | -1.53 | 4.87 | 1.11 | 0.48 | 8.56 | -5.71 | 3.91 |
|  | SE | 0.43 | 1.15 | 1.07 | 0.38 | 1.03 | 0.97 | 0.31 | 0.73 | 0.61 | 0.41 | 0.70 | 0.63 |
| **4-class model** |  |  |  |  |  |  |  |  |  |  |  |  |  |
| Low symptoms (59.4%) | Estimate | 1.95 | -1.18 | 2.11 | 2.03 | -0.01 | 0.83 | 1.98 | -2.15 | 2.54 | 4.55 | -6.80 | 5.05 |
|  | SE | 0.04 | 0.13 | 0.14 | 0.04 | 0.14 | 0.14 | 0.04 | 0.10 | 0.10 | 0.11 | 0.13 | 0.12 |
| Moderate symptoms in children (22.6%) | Estimate | 3.30 | -0.41 | 2.32 | 2.50 | -0.10 | 0.95 | 3.64 | 0.20 | 1.63 | 8.52 | -4.51 | 2.87 |
|  | SE | 0.13 | 0.44 | 0.37 | 0.09 | 0.33 | 0.30 | 0.19 | 0.61 | 0.43 | 0.23 | 0.59 | 0.48 |
| Notable symptoms in fathers (10.3%) | Estimate | 3.60 | -0.29 | 2.08 | 7.52 | 6.98 | -5.13 | 3.03 | -2.32 | 3.29 | 6.35 | -7.40 | 5.57 |
|  | SE | 0.25 | 0.56 | 0.53 | 0.28 | 1.00 | 0.90 | 0.12 | 0.36 | 0.35 | 0.19 | 0.47 | 0.42 |
| Co-occurring maternal and child symptoms (7.7%) | Estimate | 10.24 | 1.67 | -0.74 | 4.29 | 2.88 | -0.76 | 5.19 | 2.47 | -0.61 | 9.14 | -4.52 | 2.73 |
|  | SE | 0.77 | 1.34 | 1.14 | 0.26 | 1.09 | 1.06 | 0.25 | 1.22 | 0.93 | 0.35 | 1.15 | 0.96 |

*Note*. *Based on estimated posterior probabilities.

**Table S16: Relations of the identified trajectory groups to outcomes of self-harm and suicide attempts in the sample with biological parents**

|  | Outcome means (SE) by class | | | | | | Wald test *p* value | | | | | |
| --- | --- | --- | --- | --- | --- | --- | --- | --- | --- | --- | --- | --- |
| **3-class model** | Low symptoms (c1) | | Notable externalizing problems in children (c2) | | Co-occurring maternal and child symptoms (c3) | | c1 vs. c2 | c1 vs. c3 | c2 vs. c3 |  |  |  |
| Age14 self-harm | .13 (.01) ^2,3^ | | .15 (.01) ^1^ | | .18 (.02) ^1^ | | .013* | .001** | .143 |  |  |  |
| Age17 self-harm | .21 (.01) ^2,3^ | | .25 (.01) ^1^ | | .26 (.02) ^1^ | | .001** | .008** | .694 |  |  |  |
| Lifetime suicide attempts (reported at age 17) | .05 (.00) ^2,3^ | | .09 (.01) ^1,3^ | | .13 (.02) ^1,2^ | | <.001*** | <.001*** | .014* |  |  |  |
| **4-class model** | Low symptoms (c1) | Moderate symptoms in children (c2) | | Notable symptoms in fathers (c3) | | Co-occurring maternal and child symptoms (c4) | c1 vs. c2 | c1 vs. c3 | c1 vs. c4 | c2 vs. c3 | c2 vs. c4 | c3 vs. c4 |
| Age14 self-harm | .13 (.01) ^2,4^ | .16 (.01) ^1,3^ | | .12 (.01) ^2,4^ | | .19 (.02) ^1,3^ | .015* | .702 | <.001*** | .046* | .060† | .001** |
| Age17 self-harm | .21 (.01) ^2,3^ | .26 (.01)^1^ | | .26 (.02)^1^ | | .24 (.02) | .001** | .009** | .097† | .969 | .570 | .591 |
| Lifetime suicide attempts (reported at age 17) | .05 (.00) ^2,3,4^ | .08 (.01) ^1,4^ | | .09 (.01) ^1,4^ | | .13 (.02) ^1,2,3^ | <.001*** | .002** | <.001*** | .979 | .014* | .026* |

*Note.* Numbers in superscript refers to significantly different subgroups in the outcomes. P values indicate for the Wald tests.


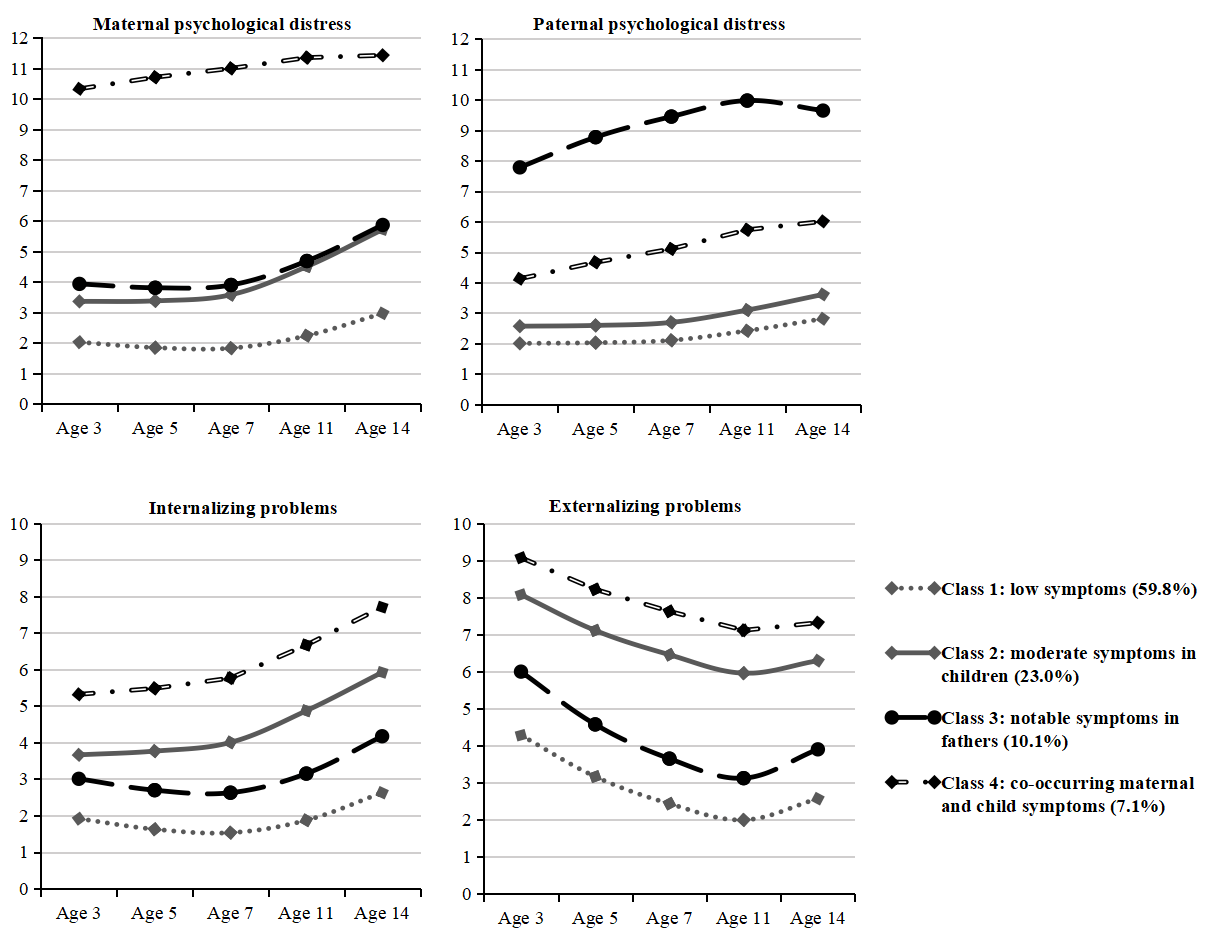


**Figure S1. Joint maternal and paternal psychological distress and child internalizing and externalizing problems trajectories (Female sample)**


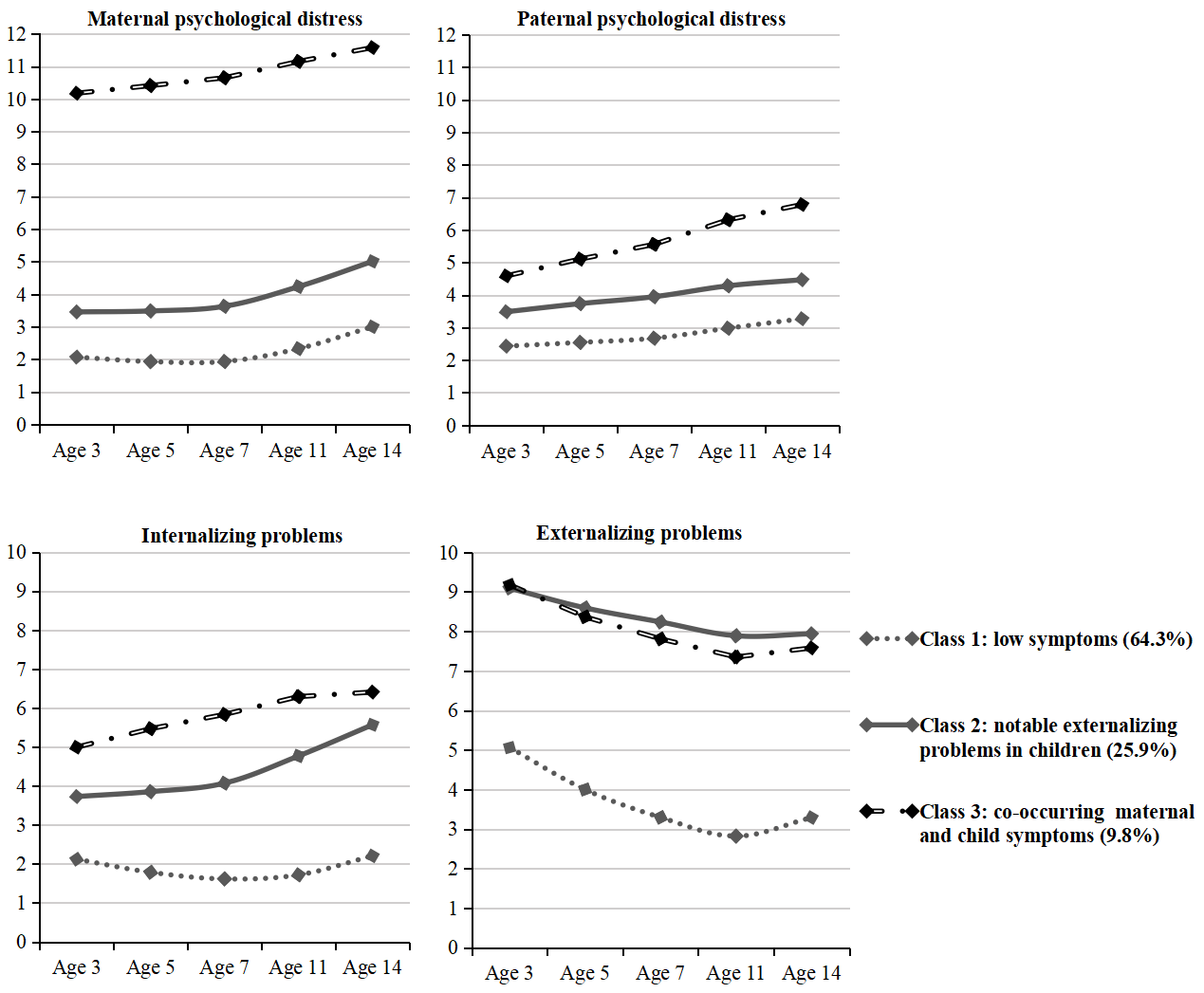


**Figure S2. Joint maternal and paternal psychological distress and child internalizing and externalizing problems trajectories (Male sample)**


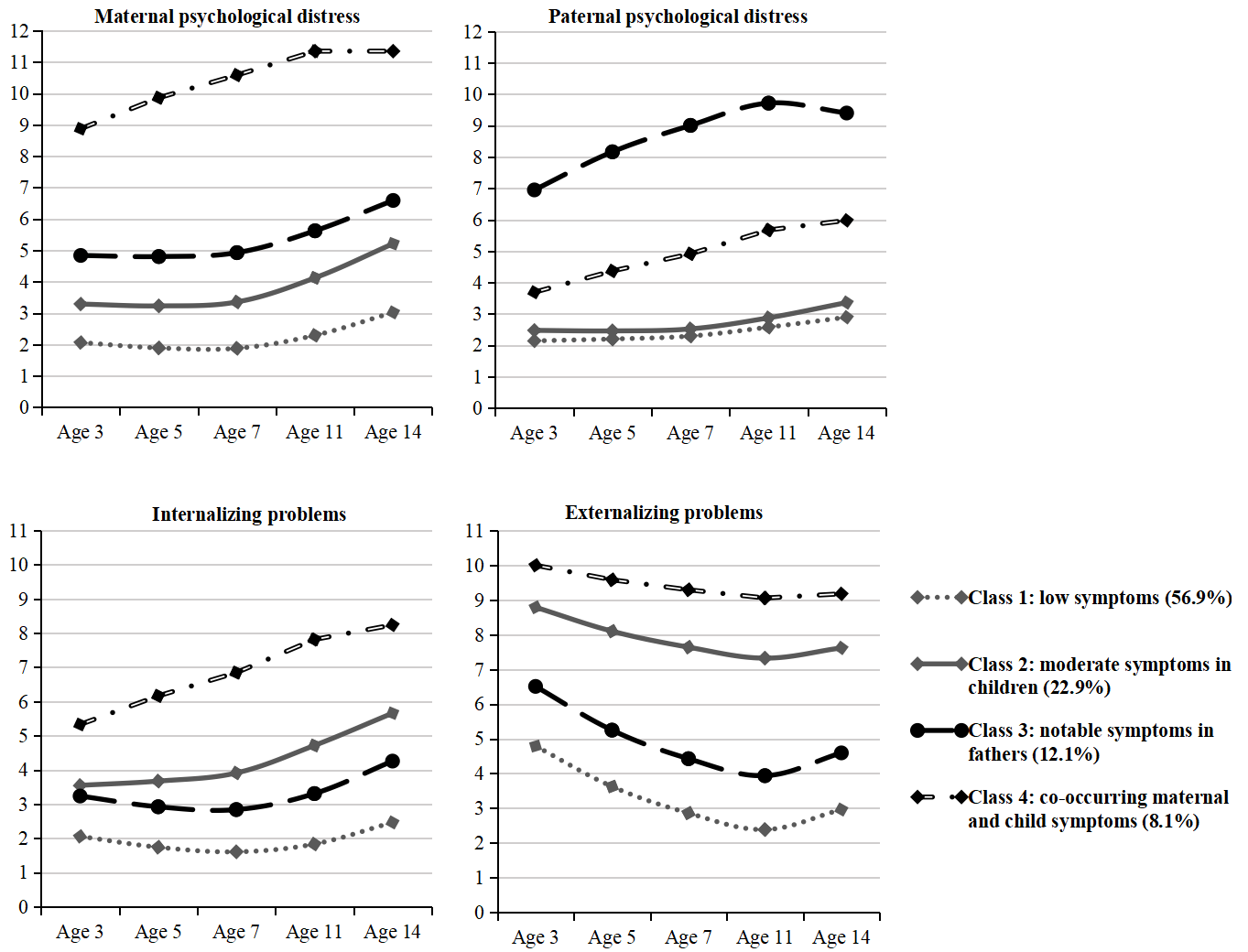


**Figure S3: 4-classes model in the whole sampe (Adjusted results)**


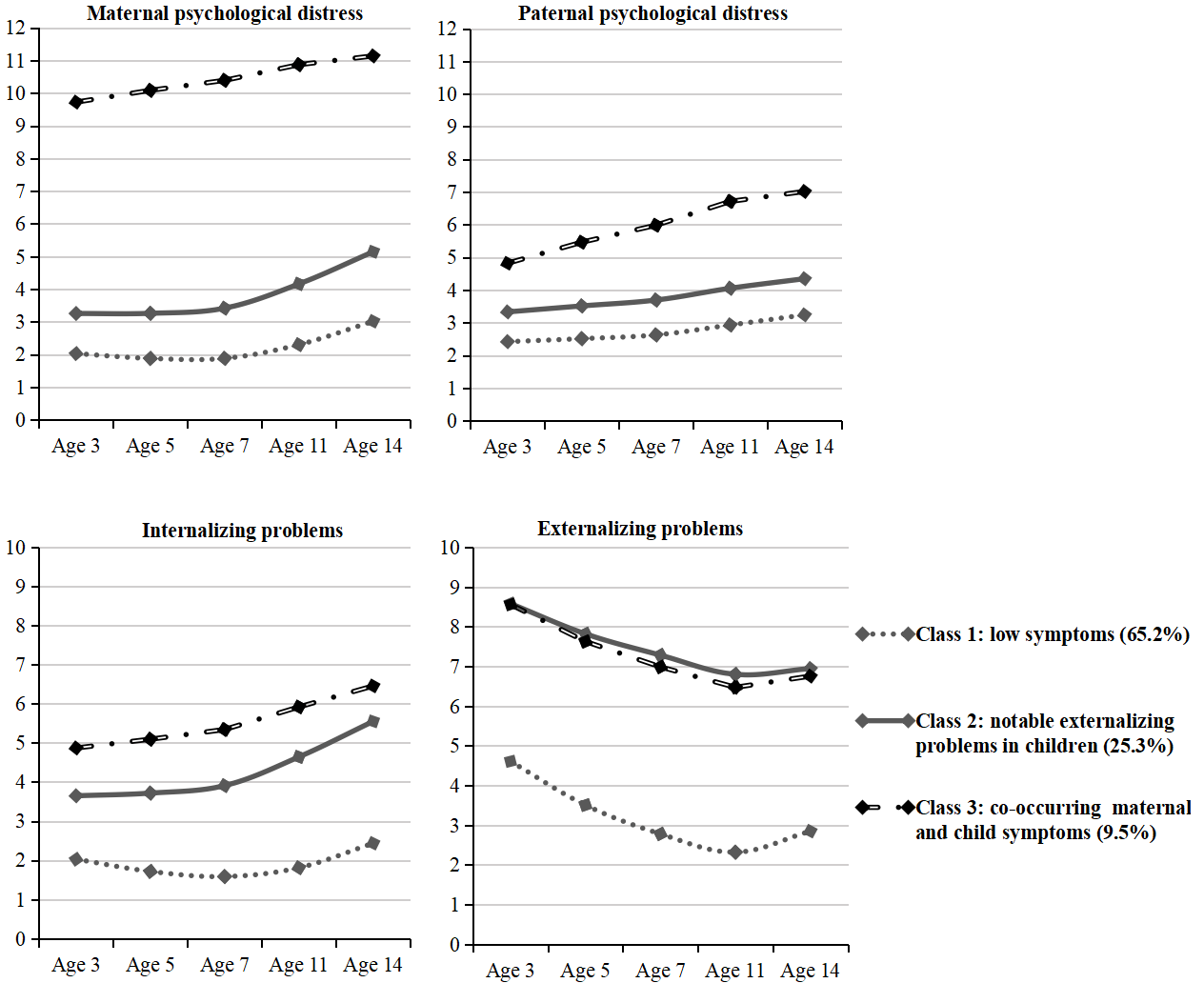


**Figure S4: 3-classes model in the sample with biological parents**


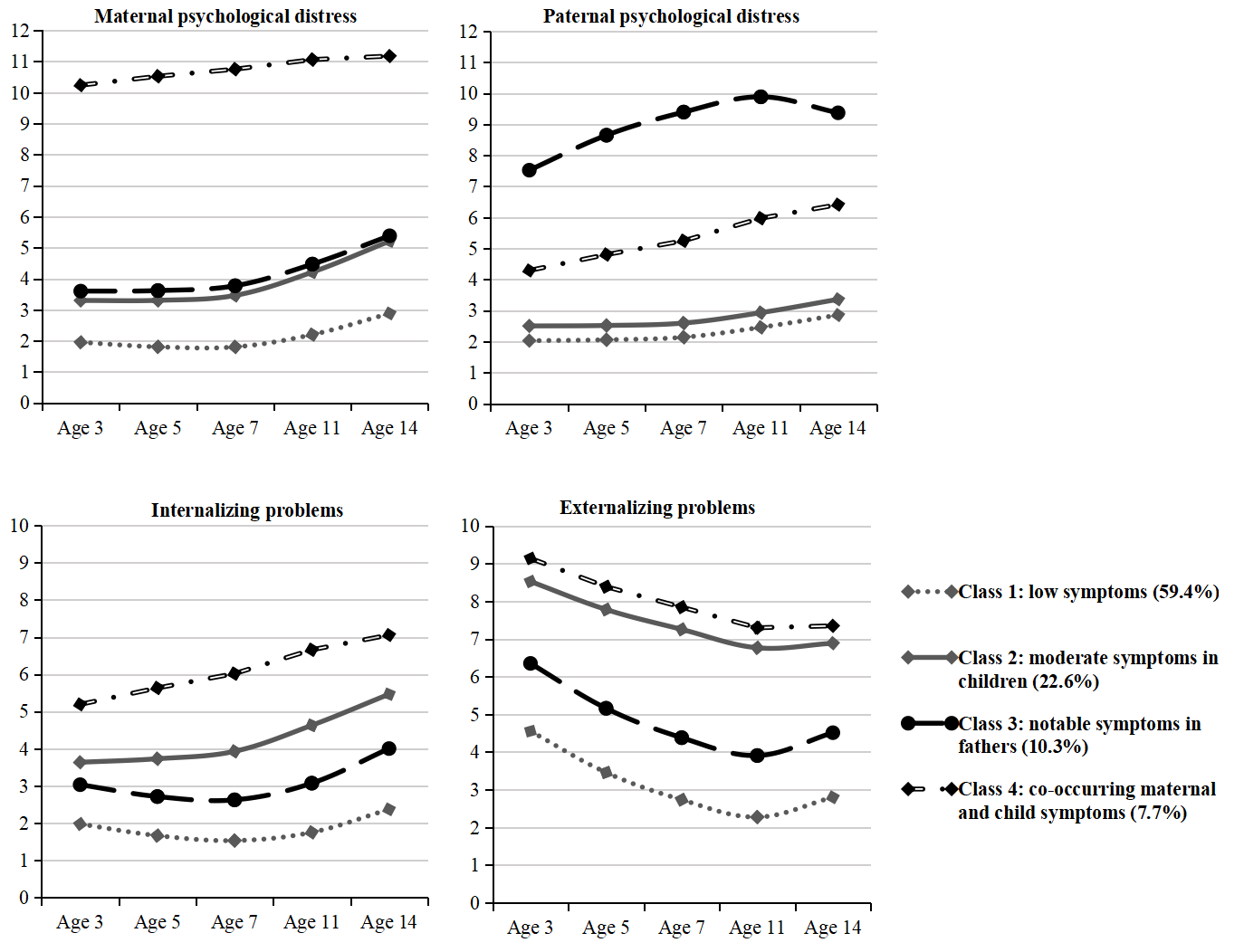
**Figure S5: 4-classes model in the sample with biological parents**
